# Histone acetyltransferase HBO1 in cancer biology: Essential mechanisms and implications for targeted therapeutics

**DOI:** 10.1016/j.bbcan.2026.189533

**Published:** 2026-01-18

**Authors:** Alissa D. Marchione, Katie L. Kathrein

**Affiliations:** Department of Biological Sciences, University of South Carolina, Columbia, SC 29208, United States of America

**Keywords:** HBO1/KAT7, Histone acetyltransferase, HBO1 complex, Oncogenesis, Targeted therapy

## Abstract

The histone acetyltransferase complex HBO1 (KAT7) is an oncogenic regulator across multiple cancers, promoting cell proliferation and migration. Though clinically important, no targeted therapies address HBO1 dysregulation. HBO1 forms complexes with MEAF6, JADE(1/2/3), ING(ING4/5), and BRPF1/2/3 to acetylate histones H3 and H4, especially at H3K14, promoting transcriptional activation and genomic stability. It colocalizes with active transcriptional sites and participates in gene regulation, DNA repair and replication. Most HBO1-associated cancer mutations are missense, though their effects remain unclear. Silencing HBO1 restores normal proliferation and gene expression, underscoring its oncogenic role. HBO1 activity supports cancer pathways, including apoptosis resistance, DNA damage response, and cell cycle regulation. The HBO1 inhibitor WM-3835 disrupts H3K14 acetylation, reducing tumor growth in several cancers. This review provides insights into the function of HBO1 in cancer, especially in histone acetylation, ubiquitination, stem cell maintenance, and pro- and anti-oncogenic signaling. Understanding the roles of HBO1 may guide new epigenetic therapies for HBO1-driven malignancies.

## 1. Introduction

Cancer remains a leading cause of mortality worldwide, with millions of deaths annually attributed to uncontrolled cellular growth, proliferation, and aggressive malignancy [1,2]. The molecular complexity of cancer involves distinct genetic and epigenetic alterations unique to each cancer type and even individual tumors [3–5]. Among epigenetic regulators, histone-modifying enzymes that create various post-translational modifications (PTMs) on histones have attracted growing attention as critical determinants of chromatin structure and gene expression, positioning them as promising therapeutic targets [5–7].

Histone acetylation, facilitated by histone acetyltransferases (HATs), regulates chromatin accessibility and affects various cellular processes, including transcription, DNA replication, and repair. Dysregulation of HAT activity is linked to cancer development, as abnormal acetylation patterns can lead to increased oncogene expression and tumor growth [7,8]. HBO1 (Histone Acetyltransferase Binding to ORC1, also known as KAT7) is a MYST family HAT that acetylates histone H3 and H4 at specific lysine residues, playing a crucial role in chromatin remodeling and genome stability [9–11]. HBO1 forms a complex with cofactors such as JADE1/2/3, BRPF1/2/3, ING4/5, which determine substrate specificity and functional output [12–15].

Recent genomic and transcriptomic analyses have revealed that HBO1 is broadly expressed in normal tissues, with particularly high levels in the testis and ovary; however, it is frequently overexpressed or amplified in various cancers [16–19]. Elevated expression is associated with enhanced tumor cell proliferation, migration, and poor clinical outcomes, marking HBO1 as a potential oncogenic driver and therapeutic target [7,18–20]. Despite this, the detailed molecular mechanisms by which HBO1 contributes to tumorigenesis remain largely unknown, and no targeted therapies against HBO1 are currently approved for clinical use.

## 2. Structure and function of HBO1 and the HBO1 complex

### 2.1. HBO1 structure

**Fig. 1.**
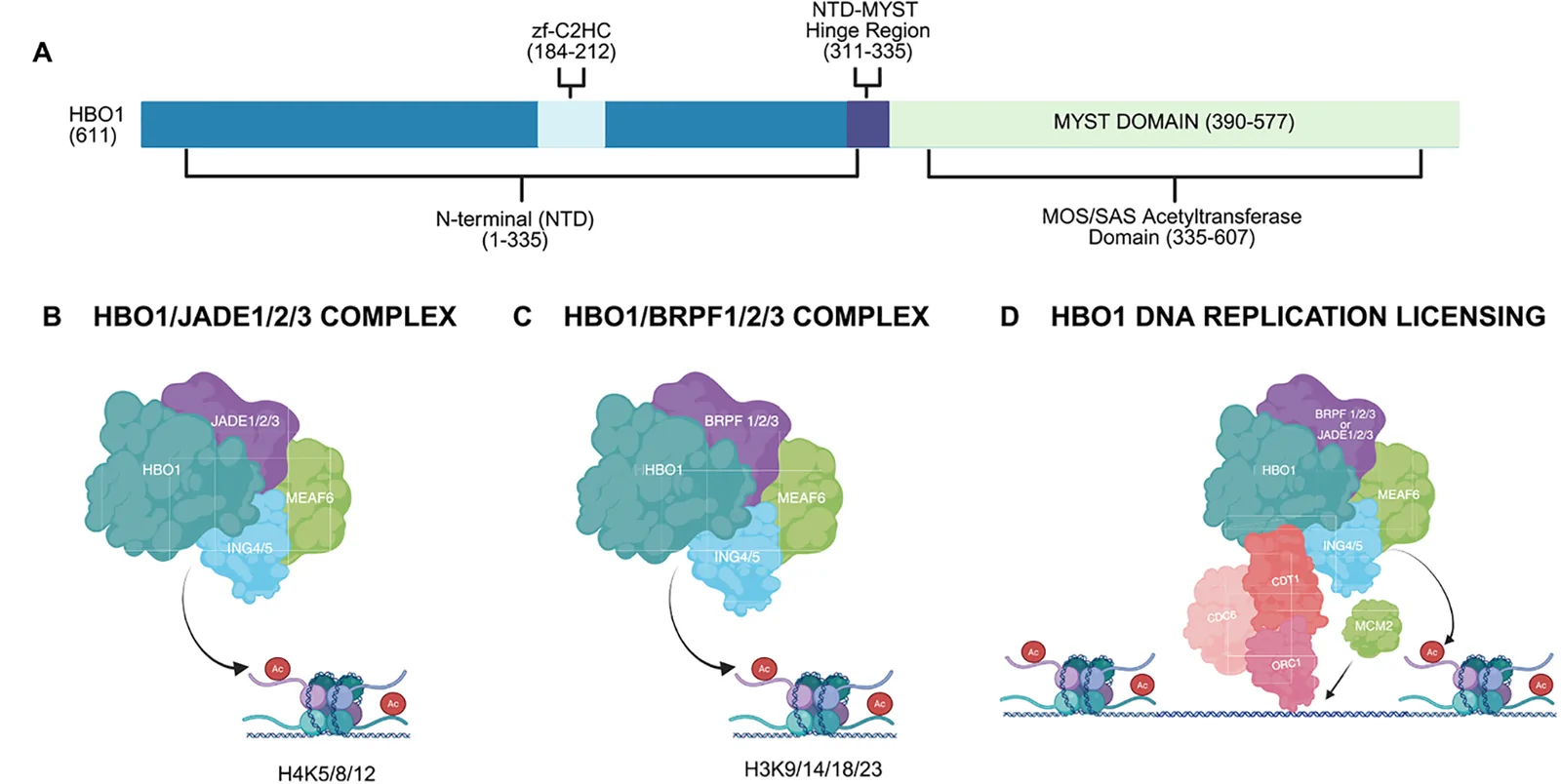
Structural Domains and Functional Complexes of HBO1 in Histone Acetylation and DNA Replication. (A) HBO1 is a MYST family lysine acetyltransferase composed of an N-terminal domain (NTD) and a catalytic MYST domain. (B) HBO1 forms complexes with JADE1, JADE2, or JADE3, which direct its acetyltransferase activity toward histone H4 at lysines 5, 8, and 12. (C) In contrast, association with BRPF1, BRPF2, or BRPF3 targets HBO1 activity toward histone H3, particularly at lysine 14. (D) During pre-replicative complex assembly in late mitosis, HBO1 binds to CDT1, acetylates the N-terminal tail of histone H4, and facilitates the chromatin loading of MCM helicase complexes, promoting DNA replication licensing. [23]

Encoded by the KAT7 (lysine acetyltransferase 7) gene, human HBO1 is a 611-amino-acid protein primarily composed of two key regions, an N-terminal domain (NTD) and a C-terminal MYST acetyltransferase domain (Fig. 1A) [9,19]. The NTD contains a short zf-C2HC-type DNA-binding motif, which enables interactions with MCM2 and ORC1 (Fig. 1A) [21]. Apart from the zf-C2HC motif, the remainder of the NTD does not correspond to any recognized domain. However, computer modeling suggests that the NTD contains multiple loops and several helices, common features among many chromatin regulatory proteins [15,19]. The C-terminal MYST domain, responsible for binding acetyl-CoA and carrying out acetylation reactions, is a conserved domain, homologous to other acetyltransferases in the MYST family, including MYST1 (MOF/KAT8) and MYST3 (MOZ/KAT6A) (Fig. 1A) [15,22]. Notably, HBO1 features a unique cervical-loop structure near the MYST domain, which facilitates the binding of cofactors to form complexes [12,15,22].

### 2.2. HBO1 and the HBO1 complex: multifunctional acetyltransferase

HBO1 is a multifunctional histone acetyltransferase (HAT) and the catalytic core of a highly dynamic tetrameric complex involved in transcriptional regulation, signaling, and cell growth [24–27]. This HBO1 complex includes three non-catalytic subunits, ING4/5 (Inhibitor of Growth 4/5), MEAF6 (MYST/Esa1-associated factor 6), and either a member of the JADE (JADE1/2/3) or BRPF (BRPF1/2/3) protein families (Fig. 1B–C) [12,14,28,29]. These scaffolding and regulatory components not only stabilize the complex but also guide substrate specificity and genomic targeting.

Historically, the HBO1-JADE and HBO1-BRPF complexes have been shown to serve distinct functions in initiating acetylation, likely due to their differing histone modification targets (Fig. 1B–C). When bound to BRPF1, HBO1 preferentially acetylates H3K14, H3K9, H3K18 and H3K23 (Fig. 1C and Table 1) [14,15,30]. BRPF proteins contain multiple domains, including PZP, bromodomain, and PWWP, allowing them to interact with modified histones [14,15,31]. BRPF3-associated complexes further enhance acetylation of H3K9, H3K14, and H4K16 [14,15,30,31]. In contrast, when HBO1 complex is formed with a JADE protein (JADE1/2/3), it targets lysines 5, 8, and 12 on histone H4 (Fig. 1C and Table 1) [12,13,26]. JADE also has PHD domains that work alongside ING4/5 to boost acetylation [12,13]. ING4 and ING5, part of the ING tumor suppressor family, are involved in regulating cell cycle and apoptosis [28,32,33]. They recognize the H3K4me3 histone mark at transcription start sites via their PHD domains, guiding the HBO1 complex to active gene regions to promote acetylation [30]. Despite advances in understanding the complex and its function, key questions remain, such as the exact nature of its interaction with MEAF6 and the differences among BRPF and JADE family proteins. While the function is not entirely understood, MEAF6 has recently been recognized as non-essential for HAT activity; however, it modulates the assembly of the HBO1 complex [27,29,34]. Further research is needed to fully elucidate these mechanisms and their biological significance.

### 2.3. HBO1 complex and gene replication licensing

DNA replication licensing is a stepwise process that involves the identification of replication origins, the assembly of pre-replicative complexes at these sites, and the initiation of replication forks [21,25,55]. This sequence of events is tightly controlled and depends on several key proteins, including ORC, CDC6, CDT1, and the MCM (minichromosome maintenance) complex [21,56]. During the assembly of the replication complex, ORC initially recruits CDC6 and CDT1 to the replication origin in the early stage of the G1 phase [55,57,58]. Subsequently, CDT1 directly interacts with the HBO1 complex, facilitating the recruitment of HBO1 to the replication origin (Fig. 1D) [57,58]. Earlier work strongly suggests that the interaction between MCM2 and HBO1 is direct and mediated by the C2HC zinc finger of HBO1 [21,57]. Although the exact mechanism by which HBO1 supports MCM loading remains unclear, it is thought to act as a bridge between CDT1 and MCMs. During cellular stress, the phosphorylation of CDT1 inhibits binding to HBO1, thereby blocking replication initiation [59,60]. These findings suggest that HBO1 functions as a co-activator of CDT1 in the DNA replication licensing process.

### 2.4. Alternate biological functions

HBO1 extends regulatory roles beyond acetylation to include transcriptional regulation and non-histone protein acyl modifications. Through the conserved MYST domain, HBO1 interacts with various nuclear receptors such as the androgen receptor (AR), progesterone receptor (PR), and co-activator SRC1, utilizing either the serine-rich N-terminal or catalytic region of the MYST domain [35–37]. Specifically, HBO1 suppresses AR- and NF-κB-driven gene expression, potentially by interfering with their access to chromatin or by recruiting repressive cofactors [36,38]. In contrast, it enhances PR-mediated transcription by promoting chromatin remodeling, likely through histone acetylation, which increases accessibility at PR target promoters [39]. HBO1 also forms alternative transcriptional complexes and has been shown to promote tissue-specific gene expression through intragenic acetylation and Pol II recruitment [40]. Recent findings suggest that HBO1 enzymatic activity extends beyond acetylation of histones H3 and H4 to include other acyl modifications such as propionylation, butyrylation, crotonylation, and H2A acetylation (Table 1) [11,20,41–44]. HBO1 is also implicated in newer histone modifications, such as lysine benzoylation (Kbz), which is associated with gene regulation and cancer and may play a role in acetoacetylation (Kacac) [7,45,46]. HBO1 functions as a non-canonical, scaffold-dependent ubiquitin ligase through its association with JADE subunits (JADE1/2/3)(Table 1) [47,48]. While JADE proteins harbor the E3 ubiquitin ligase activity, stabilization of the HBO1–JADE complex on chromatin enables JADE-mediated histone ubiquitination, coordinating DNA replication, chromatin accessibility, and transcriptional regulation [47,49]. These insights position HBO1 as a versatile regulator of chromatin structure, gene expression, and protein stability, highlighting the need for further research to unravel the functions of its complex components in health and disease.

### 2.5. Regulation of HBO1 by ubiquitination, phosphorylation, and acetylation

HBO1 activity is precisely regulated by phosphorylation, ubiquitination, and acetylation, which together modulate protein stability and enzymatic activity. Degradation of HBO1 is controlled by two E3 ubiquitin ligase complexes, CRL4/DDB2 and SCF/FBXW15, each responding to different signals [50–53]. Upon DNA damage, ATM-dependent phosphorylation at Ser50 and Ser53 triggers CRL4/DDB2-mediated ubiquitination and proteasomal degradation of HBO1, promoting cell cycle arrest and DNA repair (Table 2) [54]. In contrast, endotoxin exposure induces MEK1-dependent SCF/FBXW15 ubiquitination at Lys338, which regulates cell proliferation (Table 2) [52]. This process is balanced by USP25, which deubiquitinates and stabilizes HBO1 during inflammatory responses, thereby enhancing the transcription of inflammatory genes [61,62]. Phosphorylation of HBO1 peaks during the G2/M phase; CDK1 phosphorylates Thr85 and Thr88, creating a docking site for PLK1-mediated phosphorylation at Ser57, which enhances acetyltransferase activity, essential for DNA replication (Table 2) [59,63]. CDK11 also interacts with the N-terminal region of HBO1 by phosphorylating specific serine residues to modulate HBO1 acetyltransferase activity and coordinate replication licensing with transcriptional and apoptotic programs [55,64]. Additionally, HBO1 undergoes autoacetylation at Lys199 and the conserved Lys432 (Table 2) [65–68]. Lys432, situated near the acetyl-CoA binding site, is likely to influence enzymatic function [66,68]. Together, these coordinated modifications and interactions finely tune the stability and activity of HBO1, enabling it to play diverse roles in chromatin remodeling, DNA repair, inflammation, and cell cycle progression.

## 3. The dual role of HBO1 in cancer

HBO1 has emerged as a critical epigenetic regulator whose aberrant expression and activity are linked to cancer initiation and progression across diverse tumor types. HBO1 has oncogenic potential, which is underscored by frequent overexpression and gene amplification in malignancies such as breast, lung, gastric, prostate, ovarian, bladder, and hepatocellular carcinomas [16,18,79–85]. However, accumulating evidence suggests that HBO1 can exhibit both pro- and anti-oncogenic roles, depending on the cellular context, post-translational modifications, and interacting partners.

HBO1 function is largely dictated by cellular context and signaling pathways that are frequently hijacked in cancer. As a histone acetyltransferase, it drives the expression of genes involved in cell cycle progression, DNA replication, and oncogenesis [40,75]. Through interactions with cofactors like JADE and BRPF family proteins, HBO1 substrate specificity and chromatin targeting are fine-tuned, supporting roles in replication licensing and genome stability [14,29,32]. In ovarian cancer, for example, JADE2 enhances HBO1 acetyltransferase activity and regulates YAP1-driven mechanotransduction, affecting metastatic potential [86]. In NSCLC, HBO1 overexpression leads to elevated H3-H4 acetylation and upregulation of oncogenic targets such as CCR2, MYLK, VEGFR2, and OCIAD2, promoting proliferation and migration [80]. Conversely, HBO1 can act as a repressor through E3 ubiquitin ligase activity, notably targeting estrogen receptor alpha (ERα) for degradation in breast cancer, thereby attenuating ERα signaling and influencing tumor growth and therapy response [87,76]. This repressive function is particularly relevant in metastatic breast cancers harboring hyperactive ERα mutants, which are especially vulnerable to HBO1-mediated degradation, highlighting its complex and context-specific roles in cancer biology, further implicating HBO1 in cancer aggressiveness [87,76].

Emerging evidence also links HBO1 to cancer stem cell (CSC) biology, essential for tumor initiation, metastasis, and therapy resistance. HBO1 maintains CSC self-renewal by epigenetically regulating genes involved in proliferation and survival, thereby reinforcing key cancer hallmarks, including sustained proliferative signaling and evasion of growth suppressors [33,79,77,78,88]. Therapeutically, HBO1 inhibition using small molecules like WM-3835 has demonstrated anti-proliferative effects in NSCLC and other cancer models by disrupting histone acetylation-dependent gene expression [80,81]. These findings support the potential of HBO1 as a druggable target in epigenetically driven malignancies. Nevertheless, the ability to engage both oncogenic and tumor-suppressive pathways highlights the need for a nuanced understanding of the functional role of HBO1 within specific tumor contexts.

### 3.1. HBO1 structural mutations promote genomic instability

**Fig. 2.**
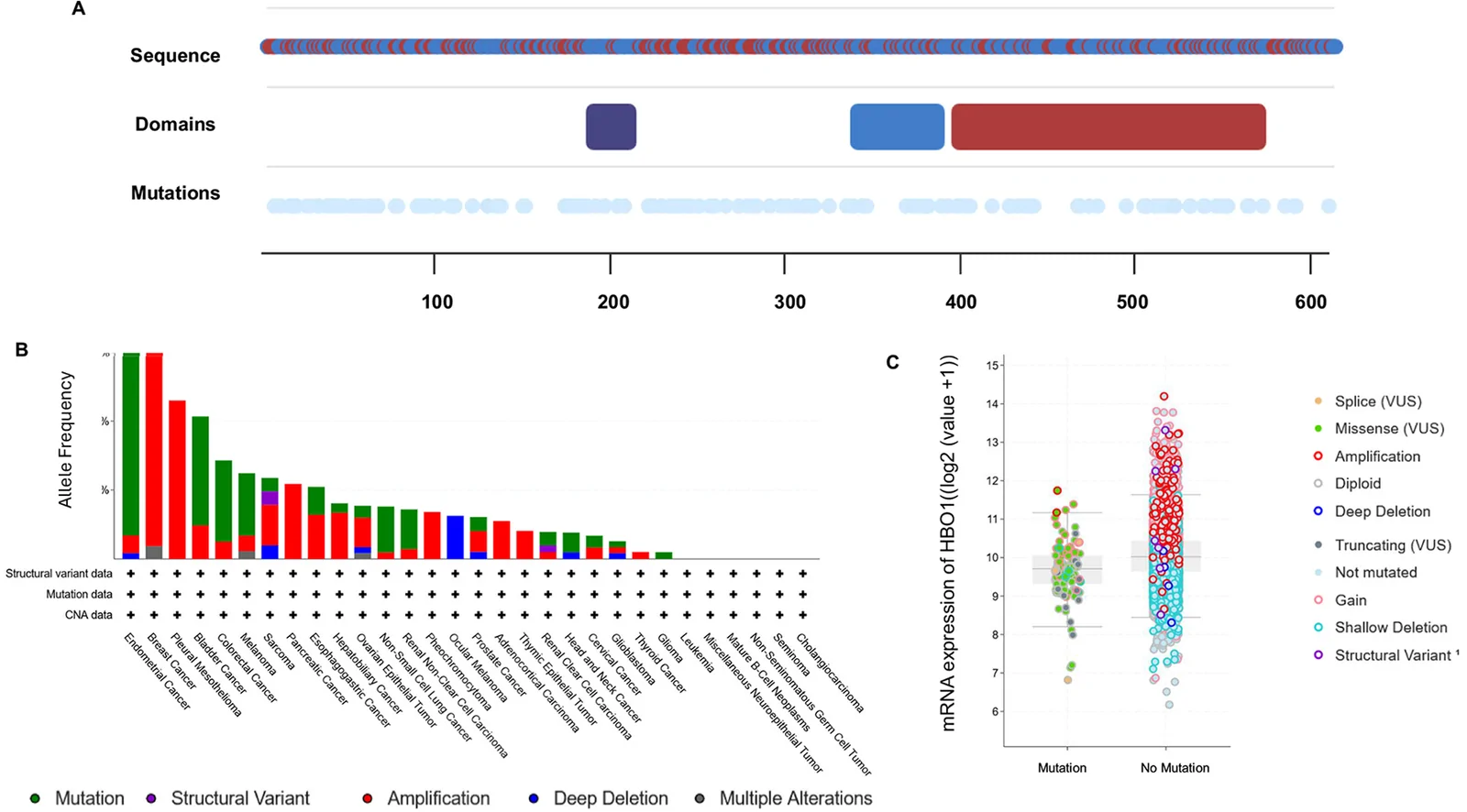
Structural alterations, mutation burden, and expression profiles of HBO1 across human cancers. (A) Schematic of HBO1 protein showing conserved domains and somatic mutations [84]. (B) Frequency of HBO1 alterations across TCGA cancer types, including mutations, amplifications, deletions, and structural variants. Endometrial and breast cancers show high alteration rates. (C) HBO1 mRNA expression by mutation category. While amplifications correlate with high expression, missense and splice mutations, especially in diploid tumors, do not, suggesting that these mutations may have expression-independent mechanisms of dysfunction [85,69,90]. *The results shown here are in whole or part based upon data generated by the TCGA Research Network: https://www.cancer.gov/tcga.

While HBO1 is frequently overexpressed in solid tumors, including hepatocellular carcinoma, non-small cell lung cancer (NSCLC), and osteosarcoma, recent studies have highlighted the oncogenic potential of structural mutations (Fig. 2A). Point mutations, splice variants, and post-translational changes can impair protein folding, domain interactions, or enzymatic activity (Fig. 2A–B). Interestingly, tumors with HBO1 mutations often lack accompanying overexpression or gene amplification, unlike wild-type HBO1 tumors (Fig. 2C) [84,69,70,89]. In prostate cancer, 15–20% of tumors have amplifications disrupting HBO1 functions, causing destabilization, altered chromatin targeting, and disrupted acetyltransferase coordination with replication origin licensing [19,72,73]. Endometrial cancers also have high HBO1 mutation rates without overexpression, revealing gaps in understanding how these mutations drive tumorigenesis (Fig. 2B) [85,69,89].

Nevertheless, several studies suggest a mechanism for how mutations may alter HBO1 function. HBO1 regulates over 9000 genes involved in genome maintenance by controlling chromatin structure, replication, and transcription through the MYST and N-terminal domains, with structural disruption leading to replication stress and chromosomal instability [5,24]. One proposed model suggests that the HBO1 N-terminal domain (NTD) functions as a regulatory switch [15,19,25]. A hinge region connects the NTD and MYST domains, and in the resting state, these domains interact closely to suppress full enzymatic activity [15,19]. Additionally, mutations reduce chromatin affinity and hinder recruitment to replication origins, exacerbating genomic instability [25].

Targeted mutation studies in HBO1 suggest how specific regulatory mechanisms may alter HBO1 function. Introduction of a mutation, T88A, in the N-terminal domain prevents phosphorylation by cyclin E/CDK2 reducing oncogenicity in breast cancer stem cell-like cells [79]. Mutations in the MYST catalytic domain, like Lys432, impair HBO1 autoacetylation, essential for acetyl-CoA binding and histone recognition [66,68]. These mutations reduce histone H3 and H4 acetylation at key lysines, causing defective chromatin remodeling and misregulation of genes related to proliferation, EMT, and stemness [30,66,68]. The specific cancer-causing effects depend on mutation type, domain involved, and cellular environment. Future studies are needed to determine if these mutations act through dominant-negative mechanisms, impair chromatin recruitment, or disrupt replication checkpoint coordination. Gaining insight into these mechanisms is vital for developing targeted therapies for HBO1-related cancers.

### 3.2. HBO1-MLL axis drives epigenetic regulation of leukemia stemness

**Fig. 3.**
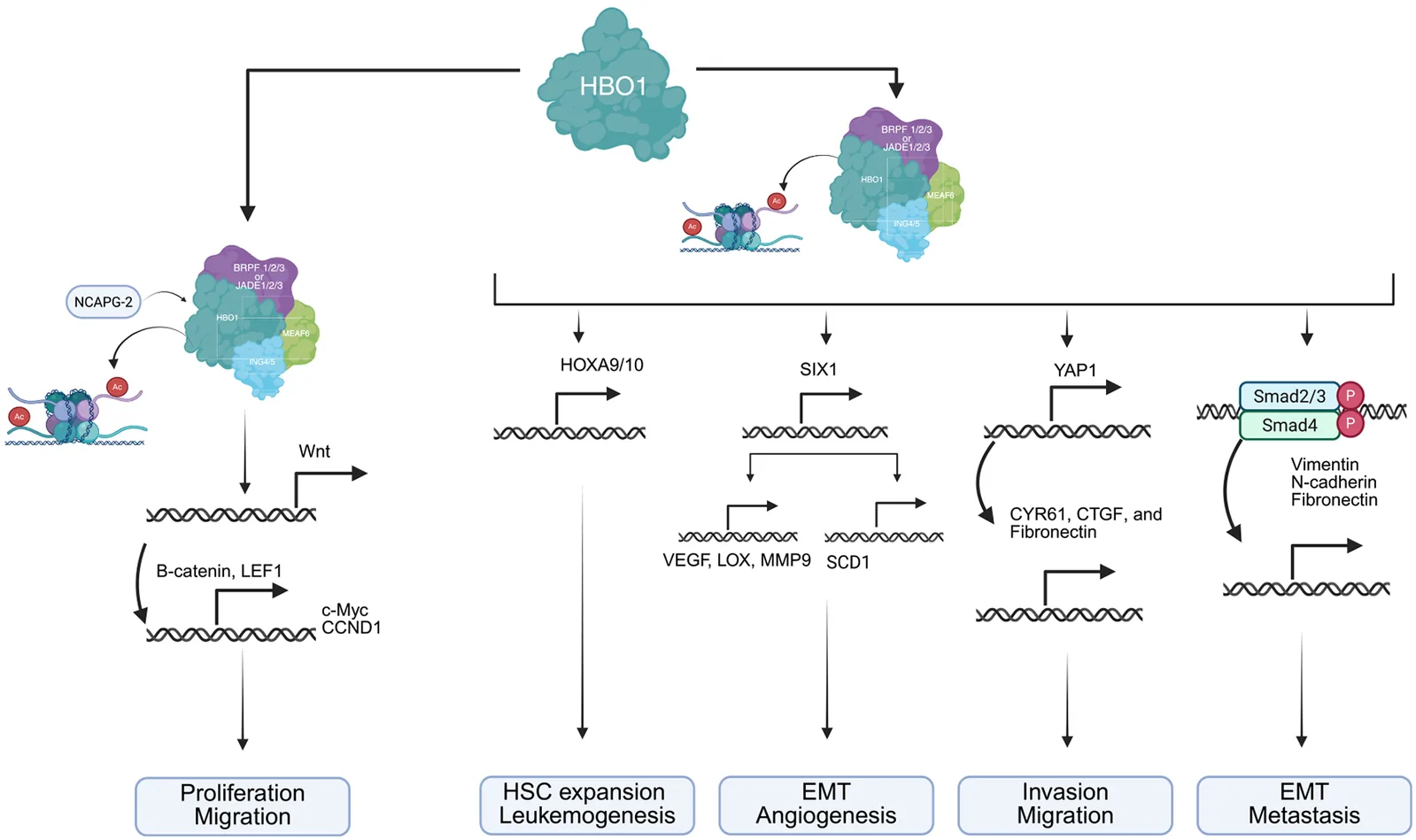
Mechanistic Overview of HBO1-Mediated Pathways in Cancer Hallmark Regulation. This figure illustrates the diverse oncogenic signaling pathways involving the histone acetyltransferase HBO1 and its associated complexes in cancer. Through histone acetylation at key promoters, including H4K5/8/12 and H3K14, HBO1 enhances the expression of Wnt signaling targets such as β-catenin, LEF1, c-Myc, and CCND1, thereby driving cellular proliferation and migration. At the HOXA9/10 loci, HBO1 activity supports the expansion of hematopoietic stem cells (HSCs) and leukemogenesis. Furthermore, HBO1 cooperates with the transcription factor SIX1 to activate genes like SCD1, VEGF, LOX, and MMP9, promoting epithelial-mesenchymal transition (EMT) and angiogenesis. HBO1 also regulates Hippo pathway effectors by promoting the transcription of YAP1, CYR61, CTGF, and fibronectin, thereby facilitating invasion and migration. Lastly, it partners with Smad2/3/4 to activate TGF-β target genes, including Vimentin, N-cadherin, and Fibronectin, driving EMT and metastasis. This comprehensive view underscores the central role of HBO1 in integrating epigenetic control with major oncogenic pathways, contributing to various cancer hallmarks. [23]

HBO1 plays a pivotal role in the epigenetic maintenance of leukemogenic programs in acute myeloid leukemia (AML) [74,71]. Regulated by scaffold subunit BRPF2, HBO1 catalyzes the acetylation of histone H3 at lysine 14 (H3K14ac), a critical modification that sustains the expression of leukemia stem cell (LSC)-associated genes, including HOXA9 and HOXA1, which are drivers of self-renewal and leukemic transformation (Fig. 3) [91,92]. This acetylation mark serves as a chromatin docking site for MLL (KMT2A) fusion-associated cofactors such as BRD4 and AF4, anchoring transcriptional complexes that maintain the oncogenic state in MLL–AF9–driven leukemias [93,94]. HBO1 also interacts directly with the MLL complex via the THD2 domain, which is conserved across all oncogenic MLL fusion proteins, enabling co-occupancy at target gene promoters [93]. This interaction facilitates the co-deposition of H3K14ac by HBO1 and H3K4me3 by MLL, thereby reinforcing a chromatin landscape that is permissive to the sustained transcription of leukemogenic targets [71,93].

Functional studies in murine and human AML models demonstrate that genetic ablation of HBO1 disrupts LSC transcriptional programs, impairs proliferation, and induces apoptosis, confirming an essential role in MLL-rearranged leukemogenesis [88,71,95]. Additionally, in chronic myelomonocytic leukemia (CMML), the NUP98–HBO1 fusion enhances H3/H4 acetylation and aberrantly activates *HOXA9*, further emphasizing the oncogenic potential of HBO1-driven acetylation beyond canonical MLL fusions [91,92]. Altogether, the HBO1–MLL axis defines a crucial epigenetic pathway in AML, wherein HBO1 acts not merely as a catalytic cofactor but as an integrative hub linking histone acetylation, transcriptional elongation, and MLL fusion–mediated oncogenesis. These findings position HBO1 as a compelling therapeutic target for disrupting core transcriptional circuitry in MLL-rearranged AML and related leukemias.

### 3.3. HBO1 drives proliferation and survival through wnt/β-catenin pathway activation

HBO1 has also emerged as a central epigenetic regulator that activates Wnt/β-catenin signaling across multiple cancer types. In glioblastoma multiforme (GBM), HBO1 function is critically regulated by phosphorylation via NCAPG2, a subunit with emerging oncogenic roles [96,97]. NCAPG2-mediated phosphorylation enhances HBO1 acetyltransferase activity, enabling upregulation of Wnt/β-catenin pathway components such as *β-catenin*, *TCF/LEF1*, and downstream effectors like *c-Myc* and *CCND1*(Fig. 3) [97,98]. This results in a chromatin state conducive to oncogenic transcription, supporting the proliferation, migration, and maintenance of GBM cells and glioma stem cells [97–99]. NCAPG2-driven HBO1 activation accelerates tumor growth, while knockdown of either gene suppresses progression, highlighting this axis as a therapeutic target [98–102]. In bladder cancer, HBO1 plays a similarly pivotal role in enhancing canonical Wnt signaling [83]. Overexpression of HBO1 increases β-catenin/TCF transcriptional activity, leading to the upregulation of proliferation-associated genes such as *c-Myc*, *LEF1*, and *CCND1* [83,103].

Mechanistically, HBO1 acetylates histones at the promoters of Wnt-responsive genes, promoting chromatin accessibility and transcription factor binding [104]. Functional studies demonstrate that HBO1 knockdown reduces Wnt target gene expression and inhibits cell proliferation, underscoring its role as a critical upstream modulator of the Wnt pathway in urothelial malignancies [83]. Xenograft models of both GBM and bladder cancer confirm that HBO1 overexpression enhances tumor growth, whereas its silencing impairs tumorigenicity [98]. In both contexts, HBO1 enables transcriptional plasticity and oncogene activation by modifying chromatin at key genomic loci. This process is either enhanced by upstream kinase signaling (NCAPG2 in GBM) or driven by overexpression (in bladder cancer). Together, these findings position HBO1 as a shared epigenetic enabler of Wnt/β-catenin-driven oncogenesis, integrating chromatin architecture, transcriptional regulation, and stemness signaling across distinct tumor types.

### 3.4. HBO1 coordinates EMT-linked invasion and metastasis through chromatin modulation

Epithelial-mesenchymal transition (EMT) is a crucial mechanism by which cancer cells acquire migratory and invasive properties, facilitating metastasis [105]. Recent studies have identified HBO1 as a key epigenetic regulator of both EMT and immune resistance in ovarian cancer [82,106,107]. During TGF-β-induced EMT, HBO1 expression is upregulated, enabling chromatin remodeling that supports transcriptional programs associated with the mesenchymal transition [82,108]. HBO1 acetylates histone H3 and H4 tails, particularly H4K5ac, H4K8ac, and H4K12ac, at gene loci involved in EMT, which promotes an open chromatin state, thereby enhancing the expression of mesenchymal markers, including vimentin, fibronectin, and N-cadherin [86,107]. Notably, the loss of H4K16ac in mesenchymal cells has been proposed as a distinguishing epigenetic feature of EMT, underscoring the biological relevance of specific histone acetylation marks [86,105]. Dysregulation of HBO1 may therefore reprogram the chromatin landscape in a manner that favors EMT and metastasis. When silenced in the ovarian cancer cell lines SKOV3, there is a reduction in EMT marker expression, impaired migratory and invasive capacity, and significantly diminished tumorigenicity in xenograft models [109]. HBO1 also cooperates with SMAD4, a key effector of the TGF-β pathway, to regulate EMT-associated gene expression [110]. This interaction stabilizes SMAD4-dependent transcriptional complexes at target promoters, further amplifying mesenchymal gene programs (Fig. 3) [110]. Together, these findings position HBO1 as a critical chromatin-modifying enzyme that links epigenetic reprogramming, epithelial-to-mesenchymal transition (EMT), and immune resistance in ovarian cancer.

### 3.5. Metabolic regulation and tumor angiogenesis by HBO1 recruitment of SIX1

HBO1 acts as a key epigenetic coactivator in oncogenic processes, particularly through an interaction with the transcription factor SIX1, which drives EMT and angiogenesis in solid tumors [111,112]. SIX1 promotes transcriptional programs that enhance cell plasticity, migration, and vascular remodeling [112–116]. HBO1 facilitates these effects by catalyzing H4K5 acetylation at SIX1 target promoters, enhancing chromatin accessibility and transcriptional activation (Fig. 3) [10,111]. It also mediates SIX1-dependent induction of *SCD1*, a lipogenic enzyme associated with mesenchymal maintenance and cancer cell plasticity [116]. In hepatocellular carcinoma cells, HBO1 loss abolishes SIX1-driven *SCD1* expression, an effect reversible with HBO1 re-expression, highlighting the necessity in this transcriptional axis [113].

In tumor angiogenesis, HBO1 regulates the expression of *VEGFR-2*, a key endothelial receptor, by maintaining RNA polymerase II occupancy and histone acetylation (H3K14 and H4) at intragenic regions of the gene [40]. HBO1-deficient zebrafish embryos exhibit vascular instability and impaired angiogenesis, rescued by human HBO1 expression [40]. In colorectal cancer, SIX1 overexpression upregulates angiogenic and metastatic factors, including *VEGF*, *LOX*, and *MMP9*, resulting in denser vascular networks [114,117]. HBO1 likely amplifies these effects by supporting transcription initiation and elongation at angiogenic loci and may also modulate transcription factor function through non-histone acetylation [40,111,118]. Together, HBO1 and SIX1 cooperate to drive chromatin remodeling programs that promote EMT and angiogenesis, linking metabolic reprogramming, epigenetic control, and oncogenic transcriptional activation.

### 3.6. YAP1 signaling driven by HBO1 promotes invasiveness in a stiffened tumor microenvironment

HBO1 has emerged as a critical epigenetic regulator of cellular mechanics and metastatic potential in ovarian and gastric cancer, modulating the Yes-associated protein 1 (YAP1) signaling axis [86,119]. YAP1, a key effector of the Hippo pathway, acts as a mechanotransducer that responds to extracellular matrix (ECM) stiffness and cytoskeletal tension by regulating genes involved in cell proliferation, survival, and migration [120,121]. HBO1 enhances YAP1 expression through direct acetylation of histone H4 at YAP1 target loci, a process facilitated by coregulator JADE2 (Fig. 3) [86]. In ovarian cancer models, HBO1-driven YAP1 upregulation promotes a mesenchymal phenotype and alters cellular mechanical properties, notably increasing elasticity and deformability, traits essential for invasive behavior and traversal of the ECM [86]. Elevated YAP1 induced by HBO1 enhances expression of downstream pro-metastatic targets such as *CYR61*, *CTGF*, and *Fibronectin*, which contribute to ECM remodeling, adhesion dynamics, and cytoskeletal reorganization (Fig. 3) [86,120,122]. These changes enhance the migratory and invasive capabilities of cancer cells, supporting their adaptation to mechanical stress within the tumor microenvironment. Abnormal HBO1-YAP1 signaling has also been associated with therapy resistance and poor clinical outcomes in ovarian cancer patients, underscoring the relevance as a prognostic marker and therapeutic target [86,122,123]. Together, these findings position HBO1 as a master regulator of the cancer cell mechano-phenotype via epigenetic activation of YAP1. By linking histone acetylation to mechanical adaptability, HBO1 enables cancer cells to acquire traits necessary for metastasis.

### 3.7. HBO1 regulates erα degradation to suppress proliferation and survival

**Fig. 4.**
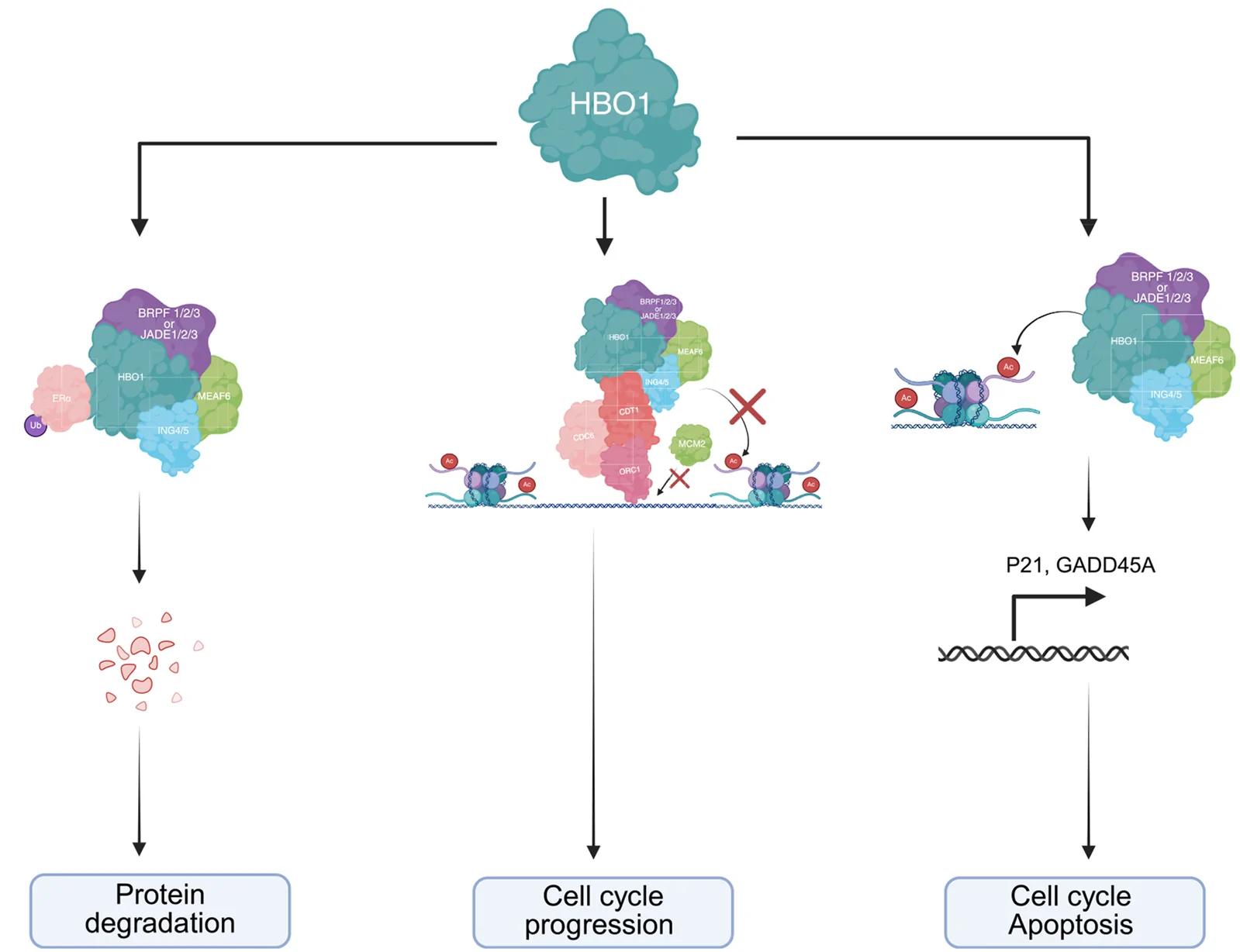
Schematic overview of HBO1-mediated regulation of tumor-suppressive signaling pathways. This figure illustrates the diverse oncogenic tumor-suppressive signaling pathways involving the histone acetyltransferase HBO1 and its associated complexes in cancer. HBO1 promotes estrogen receptor α (ERα) ubiquitination and degradation, facilitating protein turnover, and interferes with p53-regulated transcription to promote cell cycle progression. This underlines the role of HBO1 as a key epigenetic regulator of tumor-suppressive mechanisms. [23]

HBO1 plays an essential tumor-suppressive role in ERα-positive breast cancer by regulating both the activation and degradation of estrogen receptor alpha (ERα), a critical factor in hormone-driven tumor growth [87,76]. The conserved MYST domain enables HBO1 to function as a rare E3 ubiquitin ligase, targeting ERα, including hyperactive mutants common in metastatic and therapy-resistant cancers, for ubiquitination and proteasomal breakdown (Fig. 4) [87,76]. This degradation activity directly opposes proliferative signaling and is essential for maintaining hormone responsiveness. Functional studies show that HBO1 knockdown significantly increases ERα protein levels, enhances estrogen-induced transcriptional programs, and accelerates cell proliferation, while HBO1 overexpression suppresses ERα signaling and reduces tumorigenic potential [87,76]. At the same time, acetyltransferase activity of HBO1 influences transcription, positioning it at a regulatory nexus where it modulates ERα activity by balancing coactivator-mediated acetylation (such as by p300) with appropriate ubiquitination [48,124]. This dual enzymatic role enables HBO1 to participate in the cyclical turnover of ERα at target gene promoters, allowing rapid yet controlled transcriptional responses to estrogen [48,87,76]. Consequently, HBO1 maintains signaling accuracy, prevents uncontrolled cell proliferation, and sustains transcriptional stability in hormone-driven breast cancer. However, questions remain about the exact mechanisms, specifically which HBO1 domains provide E3 ligase activity and whether this function is inherent or relies on cofactors.

### 3.8. HBO1-regulated pathways govern cell cycle progression and apoptosis in cancer

HBO1 plays a dual role in promoting cell cycle progression under normal conditions and enforcing apoptosis and checkpoint responses during stress [125,126]. This regulatory network involves key interactions with p53 and CDK11p58, which together fine-tune the transcription of genes controlling replication licensing, checkpoint arrest, and apoptosis [125,127]. Dysregulation of these pathways by HBO1 overexpression or functional disruption is a common feature in several types of malignancy. In ovarian and breast cancers, HBO1 hyperactivation leads to unchecked cell cycle progression and evasion of stress-induced checkpoints [79,125,128]. These tumors frequently exhibit disrupted p53-HBO1 signaling, impairing transcription of p53 target genes including p21 and other stress response genes, thereby supporting proliferative and metastatic phenotypes.

In p53 signaling, HBO1 functions as both a regulator and a target. Under cellular stress, stabilized p53 binds to and inhibits HBO1 HAT activity, preventing inappropriate origin licensing and enforcing G1 arrest (Fig. 4) [125]. In p53-deficient tumors, loss of this negative feedback leads to sustained HBO1 activity and abnormal DNA replication [125]. HBO1 also influences p53-regulated transcription by forming complexes with ING and JADE proteins to promote p21 (CDKN1A) transcription, a cyclin-dependent kinase inhibitor essential for cell cycle checkpoints [125,129,130]. Studies show that ING4/5-containing HBO1-JADE complexes enhance p53 activity at the promoters of p21 and GADD45A, further linking HBO1 to the enforcement of the G2/M checkpoint and growth inhibition [12,125,130,131]. During etoposide-induced DNA damage, p53 recruits p300 and HBO1 to the same promoters, leading to p300-mediated p53 acetylation, boosting DNA-binding ability, and facilitating histone acetylation around TSSs. Viral interference can disrupt this pathway, as seen in HTLV–1–associated T-cell leukemia, where the viral protein HBZ interacts with the HBO1 complex, inhibiting its acetylation activity. This blunts p53-mediated transcription of p21 and Gadd45a, delaying G2 arrest (Fig. 4). These findings show that HBO1 is a vital chromatin regulator that connects DNA replication licensing with p53-dependent checkpoints and cell death pathways. When this balance is disturbed by excessive HBO1 activity, loss of normal control, or viral interference, the cell cycle becomes disconnected from genome protection. This allows replication stress and cancer growth to occur, making HBO1 and its related complexes strong candidates for new cancer treatments.

### 3.9. novel epigenetic regulation of cancer through HBO1-dependent histone lactylation

Histone lactylation (Kla) is an emerging post-translational modification that involves adding a lactyl group to lysine residues on histones, significantly influencing gene expression in cancer [132–134]. HBO1, traditionally known for acetyltransferase activity, also catalyzes histone lactylation at key sites such as H3K9la, especially at transcriptional start sites (TSSs) [135]. Lactylation is enriched at promoters/enhancers of glycolysis or metabolism-responsive genes, rather than being a global mark like acetylation [134,136]. While acetylation provides a general permissive chromatin state, this modification conveys metabolic context, gene specificity, and dynamic regulation, allowing cells to link glycolytic flux to targeted transcriptional programs that drive proliferation, survival, and immune evasion [134,137]. Studies in cancer cell lines, such as HeLa, HepG2, and MDA-MB-231, have shown that knocking out HBO1 results in a marked decrease in H3K9la levels, which in turn suppresses cancer cell proliferation, migration, and invasion [135]. Notably, the loss of H3K9la reduces cancer cell invasiveness, emphasizing the role in malignant behavior [135]. Furthermore, clinical samples from cervical cancer patients show higher HBO1 and H3K9la levels compared to nearby normal tissue [135]. These findings suggest that HBO1-mediated histone lactylation is a crucial epigenetic mechanism driving tumorigenesis and may hold promise as a prognostic marker and therapeutic target. In the broader context of precision oncology, identifying specific epigenetic biomarkers like histone lactylation is invaluable for early detection, risk assessment, treatment guidance, monitoring therapeutic response, and predicting clinical outcomes in cancer [134].

## 4. Approaches in tumor therapy and their challenges

Elevated HBO1 levels are linked to aggressive, metastatic, and treatment-resistant tumors, emphasizing the role in cancer epigenetics. Currently, therapy-induced Plk1–HBO1 signaling complicates treatment further by promoting chemoresistance [138]. In aggressive cancers, such as pancreatic ductal adenocarcinoma (PDAC), Plk1 phosphorylates HBO1, causing a shift from replication origins to activating survival genes, including cFos, which upregulates MDR1 to promote drug efflux [138]. This highlights the urgent need for targeted therapies that inhibit HBO1 activity, phosphorylation, or chromatin binding.

Strategies to counter these phenotypes include silencing the gene or inhibiting HAT activity, which then impedes gene expression related to proliferation, stemness, EMT, angiogenesis, and drug resistance. Despite the clinical success of HDAC inhibitors, the development of HAT inhibitors lags. The small molecule WM-3835 (N′-(4-fluoro-5-methyl-[1,1′-biphenyl]-3-carbonyl)-3-hydroxybenzenesulfonohydrazide) is a potent HBO1-specific inhibitor that reduces H3K14 acetylation, downregulates HOXA9/HOXA10 transcription, and inhibits acute myeloid leukemia (AML) cell growth [81,88]. WM-3835 effectively inhibits cell proliferation and induces differentiation [81]. Its efficacy extends to other cancer models, including castration-resistant prostate cancer (CRPC), non-small cell lung cancer (NSCLC), and osteosarcoma. Another agent, NBP, targets HBO1 to suppress *PD-L1* expression, reversing immune evasion mechanisms in lung cancer [139]. Beyond small molecules, microRNA-548a-3p has been shown to target SIX1; however, computational predictions suggest that it binds to the 3′ UTR of HBO1, indicating a potential dual inhibitory effect via microRNA [140]. Such dual-target miRNAs may offer a more systemic method of suppressing oncogenic networks driven by HBO1.

Although druggable, targeting HBO1 is challenging due to similarity to other MYST proteins, such as Tip60 and MOZ, which risk off-target effects. Virtual modeling has identified two potential drug sites: the acetyl-CoA site, a nearby positively charged pocket, and the hinge region connecting the N-terminal domain (NTD) to the MYST domain [67,84]. Targeting the NTD–MYST hinge could disrupt allosteric regulation and cofactor recruitment, which are vital for HBO1 interaction with BRPF and JADE subunits, necessary for chromatin localization and substrate specificity. Designing peptides or molecules that could serve as competitive inhibitors of subunit interactions, preventing chromatin recruitment and tumor growth. Computational models suggest this site may offer higher specificity for HBO1, reducing off-target effects. PROTACs are an emerging class of targeted therapeutics that induce selective protein degradation by recruiting an E3 ubiquitin ligase, offering a catalytic and potentially more complete suppression of their targets than traditional inhibitors [141,142]. Developing a WM-3835–based PROTAC against HBO1 could represent a novel strategy in cancer therapy, simultaneously eliminating acetyltransferase activity and triggering rapid protein degradation. However, developing potent and selective inhibitors remains a significant challenge [141,143].

## Figures and Tables

**Table 1:** HBO1-mediated histone modifications.

PTM Class	Histone / Site	HBO1 Complex	Functional Context	References
Acetylation	H4K5ac	HBO1–JADE	DNA replication licensing	[35,36]
	H4K8ac	HBO1–JADE	Origin firing, chromatin accessibility	[13,29,35,37]
	H4K12ac	HBO1–JADE	Replication initiation, genome stability	[13,35,38,39]
	H3K14ac	HBO1–BRPF	Transcriptional activation	[14,15,31,40]
	H3K9ac	HBO1–BRPF	Active promoters	[15,31,41]
	H3K18ac	HBO1–BRPF	Gene activation	[38,42,43]
	H3K23ac	HBO1–BRPF	Transcription	[35,41]
Ubiquitination	H4 (mono-Ub)	HBO1–JADE (Requires JADE-E3 ligase activity)	Stabilizes replication licensing complexes; reinforces open chromatin	
Lactylation	H3K18la, H4K8la	HBO1–BRPF / JADE	Metabolism-linked transcription	[44–47]
Propionylation	H3K14pr, H4K5pr	HBO1–BRPF / JADE	Transcription and replication tuning	[48,49]
Butyrylation	H4K5bu, H4K12bu	HBO1–JADE (putative)	Chromatin accessibility under high acyl-CoA	[49–51]
Crotonylation	H3K18cr, H3K9cr	HBO1–BRPF (possible)	Active transcription	[48,49,52]
H2A Acetylation	H2AK5ac / H2BK12ac	HBO1–BRPF	Open chromatin states	[53,54]

**Table 2:** Post-Translational Modifications that regulate HBO1.

PTM Class	HBO1 Site(s)	Writer / Regulator	Functional Effect on HBO1	Biological Outcome	References
Phosphorylation	Ser57 Thr85/88	CDK11p58, cell-cycle kinases	Enhances HAT activity	Promotes transcription of cell-cycle and survival genes	[61,69,70,71]
	Ser50	ATM signaling	Modulates chromatin association	Couples replication stress to HBO1 regulation	[59,54]
	Ser53				
Acetylation	Lys199	HBO1 / p300	Stabilizes chromatin binding	Sustains transcriptional activity	[72–74]
(auto)	Lys432				
Ubiquitination	Sites not mapped	CRL4–DDB2	Proteasomal degradation of HBO1	UV-induced DNA damage; replication stress	[75,76,77]
	Lys338	SCF–FBXW15	Cell-cycle–regulated turnover of HBO1	S-phase progression; replication licensing control	[78]

## Data Availability

No data was used for the research described in the article.

## References

[1] Siegel RL, Miller KD, Wagle NS, Jemal A, Cancer statistics, CA Cancer J. Clin. 73 (2023) (2023) 17–48, 10.3322/caac.21763. 36633525

[2] Bray F, Ferlay J, Soerjomataram I, Siegel RL, Torre LA, Jemal A, Global cancer statistics 2018: GLOBOCAN estimates of incidence and mortality worldwide for 36 cancers in 185 countries, CA Cancer J. Clin. 68 (2018) 394–424. doi:10.3322/caac.21492. 30207593

[3] Vogelstein B, Papadopoulos N, Velculescu VE, Zhou S, Diaz LA, Kinzler KW, Cancer Genome Landscapes. https://www.science.org, 2026. 10.1126/science.1235122 PMC3749880 23539594

[4] Hanahan D, Weinberg RA, Hallmarks of cancer: the next generation, Cell 144 (2011) 646–674, 10.1016/j.cell.2011.02.013. 21376230

[5] Chakravarthi BVSK, Nepal S, Varambally S, Genomic and epigenomic alterations in Cancer, Am. J. Pathol. 186 (2016) 1724–1735, 10.1016/j.ajpath.2016.02.023. 27338107 PMC4929396

[6] Wong KM, Hudson TJ, McPherson JD, Unraveling the genetics of cancer: genome sequencing and beyond, Annu. Rev. Genomics Hum. Genet. 12 (2011) 407–430, 10.1146/annurev-genom-082509-141532. 21639794

[7] Liu R, Wu J, Guo H, Yao W, Li S, Lu Y, Jia Y, Liang X, Tang J, Zhang H, Post-translational modifications of histones: mechanisms, biological functions, and therapeutic targets, MedComm (Beijing) 4 (2023), 10.1002/mco2.292. PMC10200003 37220590

[8] Bu S, Ye T, Gao H, Song H, Zhu Y, Histone methylation and acetylation in cancer: mechanism, progression, and targets, Oncologie (2024), 10.1515/oncologie-2024-0324.

[9] Yokoyama A, Niida H, Kutateladze TG, Côté J, HBO1, a MYSTerious KAT and its links to cancer, Biochim. Biophys. Acta Gene Regul. Mech. 1867 (2024), 10.1016/j.bbagrm.2024.195045. PMC11330361 38851533

[10] Su Z, Zhang Y, Tang J, Zhou Y, Long C, Multifunctional acyltransferase HBO1: a key regulatory factor for cellular functions, Cell. Mol. Biol. Lett. 29 (2024), 10.1186/s11658-024-00661-y. PMC11566351 39543485

[11] Xiao Y, Li W, Yang H, Pan L, Zhang L, Lu L, Chen J, Wei W, Ye J, Li J, Li G, Zhang Y, Tan M, Ding J, Wong J, HBO1 is a versatile histone acyltransferase critical for promoter histone acylations, Nucleic Acids Res. 49 (2021) 8037–8059, 10.1093/nar/gkab607. 34259319 PMC8661427

[12] Siriwardana NS, Meyer R, Havasi A, Dominguez I, Panchenko MV, Cell cycle-dependent chromatin shuttling of HBO1-JADE1 histone acetyl transferase (HAT) complex, Cell Cycle 13 (2014) 1885–1901, 10.4161/cc.28759. 24739512 PMC4111752

[13] Han J, Lachance C, Daniel Ricketts M, McCullough CE, Gerace M, Black BE, Côté J, Marmorstein R, The scaffolding protein JADE1 physically links the acetyltransferase subunit HBO1 with its histone H3–H4 substrate, J. Biol. Chem. 293 (2018) 4498–4509, 10.1074/jbc.RA117.000677. 29382722 PMC5868276

[14] Feng Y, Vlassis A, Roques C, Lalonde M, González-Aguilera C, Lambert J, Lee S, Zhao X, Alabert C, Johansen JV, Paquet E, Yang X, Gingras A, Côté J, Groth A, BRPF 3-HBO 1 regulates replication origin activation and histone H3K14 acetylation, EMBO J. 35 (2016) 176–192, 10.15252/embj.201591293. 26620551 PMC4718456

[15] Tao Y, Zhong C, Zhu J, Xu S, Ding J, Structural and mechanistic insights into regulation of HBO1 histone acetyltransferase activity by BRPF2, Nucleic Acids Res. 45 (2017) 5707–5719, 10.1093/nar/gkx142. 28334966 PMC5449618

[16] Zhong W, Liu H, Deng L, Chen G, Liu Y, HBO1 overexpression is important for hepatocellular carcinoma cell growth, Cell Death Dis. 12 (2021), 10.1038/s41419-021-03818-1. PMC8155027 34039960

[17] Gao YY, Ling ZY, Zhu YR, Shi C, Wang Y, Zhang XY, Zhang ZQ, Jiang Q, Bin Chen M, Yang S, Cao C, The histone acetyltransferase HBO1 functions as a novel oncogenic gene in osteosarcoma, Theranostics 11 (2021) 4599–4615, 10.7150/thno.55655. 33754016 PMC7978299

[18] Wang Y, Chen S, Tian W, Zhang Q, Jiang C, Qian L, Liu Y, High-expression HBO1 predicts poor prognosis in gastric Cancer, Am. J. Clin. Pathol. 152 (2019) 517–526, 10.1093/ajcp/aqz065. 31247063

[19] Lan R, Wang Q, Deciphering structure, function and mechanism of lysine acetyltransferase HBO1 in protein acetylation, transcription regulation, DNA replication and its oncogenic properties in cancer, Cell. Mol. Life Sci. 77 (2020) 637–649, 10.1007/s00018-019-03296-x. 31535175 PMC11104888

[20] Li N, Hamor C, An Y, Zhu L, Gong Y, Toh Y, Guo YR, Biological functions and therapeutic potential of acylation by histone acetyltransferases, Acta Materia Medica 2 (2023) 228–254, 10.15212/AMM-2023-0010.

[21] Burke TW, Cook JG, Asano M, Nevins JR, Replication factors MCM2 and ORC1 interact with the histone acetyltransferase HBO1, J. Biol. Chem. 276 (2001) 15397–15408, 10.1074/jbc.M011556200. 11278932

[22] Kueh AJ, Dixon MP, Voss AK, Thomas T, HBO1 is required for H3K14 acetylation and Normal transcriptional activity during embryonic development, Mol. Cell. Biol. 31 (2011) 845–860, 10.1128/mcb.00159-10. 21149574 PMC3028655

[23] Kathrein K, Created in BioRender. Https://BioRender.Com/Mplfw7w, 2026.

[24] Iizuka M, Takahashi Y, Mizzen CA, Cook RG, Fujita M, Allis CD, Frierson HF, Fukusato T, Smith MM, Histone acetyltransferase Hbo1: catalytic activity, cellular abundance, and links to primary cancers, Gene 436 (2009) 108–114, 10.1016/j.gene.2009.01.020. 19393168 PMC2674512

[25] Iizuka M, Matsui T, Takisawa H, Smith MM, Regulation of replication licensing by acetyltransferase Hbo1, Mol. Cell. Biol. 26 (2006) 1098–1108, 10.1128/MCB.26.3.1098-1108.2006. 16428461 PMC1347032

[26] Havasi A, Haegele JA, Gall JM, Blackmon S, Ichimura T, Bonegio RG, Panchenko MV, Histone acetyl transferase (HAT) HBO1 and JADE1 in epithelial cell regeneration, Am. J. Pathol. 182 (2013) 152–162, 10.1016/j.ajpath.2012.09.017. 23159946 PMC3532714

[27] Avvakumov N, Lalonde M-E, Saksouk N, Paquet E, Glass KC, Landry A-J, Doyon Y, Cayrou C, Robitaille GA, Richard DE, Yang X-J, Kutateladze TG, Côté J, Conserved molecular interactions within the HBO1 acetyltransferase complexes regulate cell proliferation, Mol. Cell. Biol. 32 (2012) 689–703, 10.1128/mcb.06455-11. 22144582 PMC3266594

[28] Dantas A, Al Shueili B, Park J, Abdullah S, Bertschmann J, Krowicki H, Djamshidi M, Yang Y, Blote K, Thalappilly S, Riabowol K, Tissue-specific localization of the ING4 targeting subunit of the HBO1 histone acetyltransferase in the cytoplasm and nucleus of secretory cells, Histochem. Cell Biol. 163 (2025), 10.1007/s00418-025-02385-2. PMC12098486 40402282

[29] Gaurav N, Kanai A, Lachance C, Cox KL, Liu J, Grzybowski AT, Saksouk N, Klein BJ, Komata Y, Asada S, Ruthenburg AJ, Poirier MG, Côté J, Yokoyama A, Kutateladze TG, Guiding the HBO1 complex function through the JADE subunit, Nat. Struct. Mol. Biol. 31 (2024) 1039–1049, 10.1038/s41594-024-01245-2. 38448574 PMC11320721

[30] Saksouk N, Avvakumov N, Champagne KS, Hung T, Doyon Y, Cayrou C, Paquet E, Ullah M, Landry AJ, Côté V, Yang XJ, Gozani O, Kutateladze TG, Côté J, HBO1 HAT complexes target chromatin throughout gene coding regions via multiple PHD finger interactions with histone H3 tail, Mol. Cell 33 (2009) 257–265, 10.1016/j.molcel.2009.01.007. 19187766 PMC2677731

[31] Barman S, Roy A, Padhan J, Sudhamalla B, Molecular insights into the recognition of acetylated histone modifications by the BRPF2 bromodomain, Biochemistry 61 (2022) 1774–1789, 10.1021/acs.biochem.2c00297. 35976792

[32] Doyon Y, Cayrou C, Ullah M, Landry AJ, Côté V, Selleck W, Lane WS, Tan S, Yang XJ, Côté J, ING tumor suppressor proteins are critical regulators of chromatin acetylation required for genome expression and perpetuation, Mol. Cell 21 (2006) 51–64, 10.1016/j.molcel.2005.12.007. 16387653

[33] Kueh AJ, Bergamasco MI, Quaglieri A, Phipson B, Li-Wai-Suen CSN, Lönnstedt IM, Hu Y, Feng ZP, Woodruff C, May RE, Wilcox S, Garnham AL, Snyder MP, Smyth GK, Speed TP, Thomas T, Voss AK, Stem cell plasticity, acetylation of H3K14, and de novo gene activation rely on KAT7, Cell Rep. 42 (2023), 10.1016/j.celrep.2022.111980. 36641753

[34] Ullah M, Pelletier N, Xiao L, Zhao SP, Wang K, Degerny C, Tahmasebi S, Cayrou C, Doyon Y, Goh S-L, Champagne N, Côté J, Yang X-J, Molecular architecture of quartet MOZ/MORF histone acetyltransferase complexes, Mol. Cell. Biol. 28 (2008) 6828–6843, 10.1128/mcb.01297-08. 18794358 PMC2573306

[35] Chi Y, Huang S, Peng H, Liu M, Zhao J, Shao Z, Wu J, Critical role of CDK11p58 in human breast cancer growth and angiogenesis, BMC Cancer 15 (2015), 10.1186/s12885-015-1698-7. PMC4608324 26470709

[36] Jaiswal B, Agarwal A, Gupta A, Lysine acetyltransferases and their role in AR Signaling and prostate Cancer, Front. Endocrinol. (Lausanne) 13 (2022), 10.3389/fendo.2022.886594. PMC9428678 36060957

[37] Sharma M, Zarnegar M, Li X, Lim B, Sun Z, Androgen receptor interacts with a novel MYST protein, HBO1, J. Biol. Chem. 275 (2000) 35200–35208, 10.1074/jbc.M004838200. 10930412

[38] Vija L, Meduri G, Comperat E, Vasiliu V, Izard V, Ferlicot S, Boukari K, Camparo P, Viengchareun S, Constancis E, Dumitrache C, Lombès M, Young J, Expression and characterization of androgen receptor coregulators, SRC-2 and HBO1, during human testis ontogenesis and in androgen signaling deficient patients, Mol. Cell. Endocrinol. 375 (2013) 140–148, 10.1016/j.mce.2013.05.004. 23707616

[39] Contzler R, Regamey A, Favre B, Roger T, Hohl D, Huber M, Histone acetyltransferase HBO1 inhibits NF-κB activity by coactivator sequestration, Biochem. Biophys. Res. Commun. 350 (2006) 208–213, 10.1016/j.bbrc.2006.09.030. 16997280

[40] Georgiakaki M, Chabbert-Buffet N, Dasen B, Meduri G, Wenk S, Rajhi L, Amazit L, Chauchereau A, Burger CW, Blok LJ, Milgrom E, Lombès M, Guiochon-Mantel A, Loosfelt H, Ligand-controlled interaction of histone acetyltransferase binding to ORC-1 (HBO1) with the N-terminal transactivating domain of progesterone receptor induces steroid receptor coactivator 1-dependent coactivation of transcription, Mol. Endocrinol. 20 (2006) 2122–2140, 10.1210/me.2005-0149. 16645042

[41] Yan MS, Turgeon PJ, Man HSJ, Dubinsky MK, Ho JJD, El-Rass S, Wang YD, Wen XY, Marsden PA, Histone acetyltransferase 7 (KAT7)-dependent intragenic histone acetylation regulates endothelial cell gene regulation, J. Biol. Chem. 293 (2018) 4381–4402, 10.1074/jbc.RA117.001383. 29414790 PMC5868248

[42] Sabari BR, Zhang D, Allis CD, Zhao Y, Metabolic regulation of gene expression through histone acylations, Nat. Rev. Mol. Cell Biol. 18 (2017) 90–101, 10.1038/nrm.2016.140. 27924077 PMC5320945

[43] Dai L, Peng C, Montellier E, Lu Z, Chen Y, Ishii H, Debernardi A, Buchou T, Rousseaux S, Jin F, Sabari BR, Deng Z, Allis CD, Ren B, Khochbin S, Zhao Y, Lysine 2-hydroxyisobutyrylation is a widely distributed active histone mark, Nat. Chem. Biol. 10 (2014) 365–370, 10.1038/nchembio.1497. 24681537

[44] Tan M, Luo H, Lee S, Jin F, Yang JS, Montellier E, Buchou T, Cheng Z, Rousseaux S, Rajagopal N, Lu Z, Ye Z, Zhu Q, Wysocka J, Ye Y, Khochbin S, Ren B, Zhao Y, Identification of 67 histone marks and histone lysine crotonylation as a new type of histone modification, Cell 146 (2011) 1016–1028, 10.1016/j.cell.2011.08.008. 21925322 PMC3176443

[45] Chen Y, Sprung R, Tang Y, Ball H, Sangras B, Kim SC, Falck JR, Peng J, Gu W, Zhao Y, Lysine propionylation and butyrylation are novel post-translational modifications in histones, Mol. Cell. Proteomics 6 (2007) 812–819, 10.1074/mcp.M700021-MCP200. 17267393 PMC2911958

[46] Tan D, Wei W, Han Z, Ren X, Yan C, Qi S, Song X, Zheng YG, Wong J, Huang H, HBO1 catalyzes lysine benzoylation in mammalian cells, IScience 25 (2022), 10.1016/j.isci.2022.105443. PMC9647509 36388951

[47] Gao Y, Sheng X, Tan D, Kim SJ, Choi S, Paudel S, Lee T, Yan C, Tan M, Kim KM, Cho SS, Ki SH, Huang H, Zhao Y, Lee S, Identification of histone lysine Acetoacetylation as a dynamic post-translational modification regulated by HBO1, Adv. Sci. 10 (2023), 10.1002/advs.202300032. PMC10477889 37382194

[48] Iizuka M, Susa T, Tamamori-Adachi M, Okinaga H, Okazaki T, Intrinsic ubiquitin E3 ligase activity of histone acetyltransferase Hbo1 for estrogen receptor α, Proc. Jpn. Acad. Ser. B Phys. Biol. Sci. 93 (2017) 498–510, 10.2183/pjab.93.030. PMC5713178 28769019

[49] Iizuka M, Susa T, Takahashi Y, Tamamori-Adachi M, Kajitani T, Okinaga H, Fukusato T, Okazaki T, Histone acetyltransferase Hbo1 destabilizes estrogen receptor α by ubiquitination and modulates proliferation of breast cancers, Cancer Sci. 104 (2013) 1647–1655, 10.1111/cas.12303. 24125069 PMC7653525

[50] Borgal L, Rinschen MM, Dafinger C, Liebrecht VI, Abken H, Benzing T, Schermer B, Jade-1S phosphorylation induced by CK1α contributes to cell cycle progression, Cell Cycle 15 (2016) 1034–1045, 10.1080/15384101.2016.1152429. 26919559 PMC4889251

[51] Wu X, Yu M, Zhang Z, Leng F, Ma Y, Xie N, Lu F, DDB2 regulates DNA replication through PCNA-independent degradation of CDT2, Cell Biosci. 11 (2021), 10.1186/s13578-021-00540-5. PMC7869461 33557942

[52] Matsunuma R, Niida H, Ohhata T, Kitagawa K, Sakai S, Uchida C, Shiotani B, Matsumoto M, Nakayama KI, Ogura H, Shiiya N, Kitagawa M, UV damage-induced phosphorylation of HBO1 triggers CRL4 DDB2 -mediated degradation to regulate cell proliferation, Mol. Cell. Biol. 36 (2016) 394–406, 10.1128/mcb.00809-15. 26572825 PMC4719422

[53] Zou C, Chen Y, Smith RM, Snavely C, Li J, Coon TA, Chen BB, Zhao Y, Mallampalli RK, SCFFbxw15 mediates histone acetyltransferase binding to origin recognition complex (HBO1) ubiquitin-proteasomal degradation to regulate cell proliferation, J. Biol. Chem. 288 (2013) 6306–6316, 10.1074/jbc.M112.426882. 23319590 PMC3585065

[54] Codilupi T, CRL4 Ubiquitin Ligase Promotes Fanconi Anemia Pathway-Induced Single-Stranded DNA Signaling at Interstrand Crosslinks, n.d. 10.1186/s12885-019-6305-x PMC6833152 31690264

[55] Zong H, Li Z, Liu L, Hong Y, Yun X, Jiang J, Chi Y, Wang H, Shen X, Hu Y, Niu Z, Gu J, Cyclin-dependent kinase 11p58 interacts with HBO1 and enhances its histone acetyltransferase activity, FEBS Lett. 579 (2005) 3579–3588, 10.1016/j.febslet.2005.05.039. 15963510

[56] Iizuka M, Stillman B, Histone acetyltransferase HBO1 interacts with the ORC1 subunit of the human initiator protein, J. Biol. Chem. 274 (1999) 23027–23034, 10.1074/jbc.274.33.23027. 10438470

[57] Miotto B, Struhl K, HBO1 histone acetylase is a coactivator of the replication licensing factor Cdt1, Genes Dev. 22 (2008) 2633–2638, 10.1101/gad.1674108. 18832067 PMC2559906

[58] Saxena S, Yuan P, Kumar Dhar S, Senga T, Takeda D, Robinson H, Kornbluth S, Swaminathan K, Dutta A, A Dimerized Coiled-Coil Domain and an Adjoining Part of Geminin Interact with Two Sites on Cdt1 for Replication Inhibition tion. At the G1-S transition, the activation of cyclin-dependent kinases results in the conversion of the pre-RC to active replication forks that initiate bidirectional DNA synthesis (Bell and Dutta, 2002). Simultaneously, multiple mechanisms come into effect to prevent the reassembly of pre-RC on origins and remain in effect, 2004. 10.1016/j.molcel.2004.06.045 15260975

[59] Niida H, Matsunuma R, Horiguchi R, Uchida C, Nakazawa Y, Motegi A, Nishimoto K, Sakai S, Ohhata T, Kitagawa K, Moriwaki S, Nishitani H, Ui A, Ogi T, Kitagawa M, Phosphorylated HBO1 at UV irradiated sites is essential for nucleotide excision repair, Nat. Commun. 8 (2017), 10.1038/ncomms16102. PMC5520108 28719581

[60] Miotto B, Struhl K, JNK1 phosphorylation of Cdt1 inhibits recruitment of HBO1 histone acetylase and blocks replication licensing in response to stress, Mol. Cell 44 (2011) 62–71, 10.1016/j.molcel.2011.06.021. 21856198 PMC3190045

[61] Matsunuma R, Niida H, Ohhata T, Kitagawa K, Sakai S, Uchida C, Shiotani B, Matsumoto M, Nakayama KI, Ogura H, Shiiya N, Kitagawa M, UV damage-induced phosphorylation of HBO1 triggers CRL4 DDB2 -mediated degradation to regulate cell proliferation, Mol. Cell. Biol. 36 (2016) 394–406, 10.1128/mcb.00809-15. 26572825 PMC4719422

[62] Long C, Lai Y, Li J, Huang J, Zou C, LPS promotes HBO1 stability via USP25 to modulate inflammatory gene transcription in THP-1 cells, Biochim. Biophys. Acta Gene Regul. Mech. 2018 (1861) 773–782, 10.1016/j.bbagrm.2018.08.001. PMC6368258 30745998

[63] Zou C, Chen Y, Smith RM, Snavely C, Li J, Coon TA, Chen BB, Zhao Y, Mallampalli RK, SCFFbxw15 mediates histone acetyltransferase binding to origin recognition complex (HBO1) ubiquitin-proteasomal degradation to regulate cell proliferation, J. Biol. Chem. 288 (2013) 6306–6316, 10.1074/jbc.M112.426882. 23319590 PMC3585065

[64] Wu Z-Q, Liu X, Role for Plk1 Phosphorylation of Hbo1 in Regulation of Replication Licensing. www.pnas.org/cgi/content/full/, 2007. 10.1073/pnas.0712063105 PMC2538859 18250300

[65] Kahle AC, The Regulation and Function of Cyclin Dependent Kinase 11 (CDK11): Analysis of the CDC2L1 Promoter and Elucidation of CDK11 P58 Function during Mitosis, 2006.

[66] Xia C, Tao Y, Li M, Che T, Qu J, Protein acetylation and deacetylation: An important regulatory modification in gene transcription (review), Exp. Ther. Med. (2020), 10.3892/etm.2020.9073. PMC7444376 32855658

[67] Yuan H, Rossetto D, Mellert H, Dang W, Srinivasan M, Johnson J, Hodawadekar S, Ding EC, Speicher K, Abshiru N, Perry R, Wu J, Yang C, Zheng YG, Speicher DW, Thibault P, Verreault A, Johnson FB, Berger SL, Sternglanz R, McMahon SB, Côté J, Marmorstein R, MYST protein acetyltransferase activity requires active site lysine autoacetylation, EMBO J. 31 (2012) 58–70, 10.1038/emboj.2011.382. 22020126 PMC3252582

[68] Deng W, Wang C, Zhang Y, Xu Y, Zhang S, Liu Z, Xue Y, GPS-PAIL: prediction of lysine acetyltransferase-specific modification sites from protein sequences, Sci. Rep. 6 (2016) 39787, 10.1038/srep39787. 28004786 PMC5177928

[69] Yang Y, Kueh AJ, Grant ZL, Abeysekera W, Garnham AL, Wilcox S, Hyland CD, Di Rago L, Metcalf D, Alexander WS, Coultas L, Smyth GK, Voss AK, Thomas T, Hall E, The Histone Lysine Acetyltransferase HBO1 (KAT7) Regulates Hematopoietic Stem Cell Quiescence and Self-Renewal, 2022. 10.1182/blood.2021013954 34724565

[70] Gao J, Aksoy BA, Dogrusoz U, Dresdner G, Gross B, Sumer SO, Sun Y, Jacobsen A, Sinha R, Larsson E, Cerami E, Sander C, Schultz N, Integrative analysis of complex cancer genomics and clinical profiles using the cBioPortal, Sci. Signal. 6 (2013), 10.1126/scisignal.2004088. PMC4160307 23550210

[71] Barbosa K, Deshpande AJ, Therapeutic targeting of leukemia stem cells in acute myeloid leukemia, Front. Oncol. 13 (2023), 10.3389/fonc.2023.1204895. PMC10437214 37601659

[72] Getz G, Gabriel SB, Cibulskis K, Lander E, Sivachenko A, Sougnez C, Lawrence M, Kandoth C, Dooling D, Fulton R, Fulton L, Kalicki-Veizer J, McLellan MD, O’Laughlin M, Schmidt H, Wilson RK, Ye K, Li D, Ally A, Balasundaram M, Birol I, Butterfield YSN, Carlsen R, Carter C, Chu A, Chuah E, Chun HJE, Dhalla N, Guin R, Hirst C, Holt RA, Jones SJM, Lee D, Li HI, Marra MA, Mayo M, Moore RA, Mungall AJ, Plettner P, Schein JE, Sipahimalani P, Tam A, Varhol RJ, Gordon Robertson A, Cherniack AD, Pashtan I, Saksena G, Onofrio RC, Schumacher SE, Tabak B, Carter SL, Hernandez B, Gentry J, Salvesen HB, Ardlie K, Winckler W, Beroukhim R, Meyerson M, Hadjipanayis A, Lee S, Mahadeshwar HS, Park P, Protopopov A, Ren X, Seth S, Song X, Tang J, Xi R, Yang L, Dong Z, Kucherlapati R, Chin L, Zhang J, Todd Auman J, Balu S, Bodenheimer T, Buda E, Neil Hayes D, Hoyle AP, Jefferys SR, Jones CD, Meng S, Mieczkowski PA, Mose LE, Parker JS, Perou CM, Roach J, Yan S, Simons JV, Soloway MG, Tan D, Topal MD, Waring S, Wu J, Hoadley KA, Baylin SB, Bootwalla MS, Lai PH, Triche TJ, Van Den Berg DJ, Weisenberger DJ, Laird PW, Shen H, Cho J, Dicara D, Frazer S, Heiman D, Jing R, Lin P, Mallard W, Stojanov P, Voet D, Zhang H, Zou L, Noble M, Reynolds SM, Shmulevich I, Arman Aksoy B, Antipin Y, Ciriello G, Dresdner G, Gao J, Gross B, Jacobsen A, Ladanyi M, Reva B, Sander C, Sinha R, Onur Sumer S, Taylor BS, Cerami E, Weinhold N, Schultz N, Shen R, Benz S, Goldstein T, Haussler D, Ng S, Szeto C, Stuart J, Benz CC, Yau C, Zhang W, Annala M, Broom BM, Casasent TD, Ju Z, Liang H, Liu G, Lu Y, Unruh AK, Wakefield C, Weinstein JN, Zhang N, Liu Y, Broaddus R, Akbani R, Mills GB, Adams C, Barr T, Black AD, Bowen J, Deardurff J, Frick J, Gastier-Foster JM, Grossman T, Harper HA, Hart-Kothari M, Helsel C, Hobensack A, Kuck H, Kneile K, Leraas KM, Lichtenberg TM, McAllister C, Pyatt RE, Ramirez NC, Tabler TR, Vanhoose N, White P, Wise L, Zmuda E, Barnabas N, Berry-Green C, Blanc V, Boice L, Button M, Farkas A, Green A, MacKenzie J, Nicholson D, Kalloger SE, Blake Gilks C, Karlan BY, Lester J, Orsulic S, Borowsky M, Cadungog M, Czerwinski C, Huelsenbeck-Dill L, Iacocca M, Petrelli N, Rabeno B, Witkin G, Nemirovich-Danchenko E, Potapova O, Rotin D, Berchuck A, Birrer M, Disaia P, Monovich L, Curley E, Gardner J, Mallery D, Penny R, Dowdy SC, Winterhoff B, Dao L, Gostout B, Meuter A, Teoman A, Dao F, Olvera N, Bogomolniy F, Garg K, Soslow RA, Abramov M, Bartlett JMS, Kodeeswaran S, Parfitt J, Moiseenko F, Clarke BA, Goodman MT, Carney ME, Matsuno RK, Fisher J, Huang M, Kimryn Rathmell W, Thorne L, Van Le L, Dhir R, Edwards R, Elishaev E, Zorn K, Goodfellow PJ, Mutch D, Kahn AB, Bell DW, Pollock PM, Wang C, Wheeler D, Shinbrot E, Gordon Robertson A, Ding L, Blake Gilks C, Mardis ER, Ayala B, Chu AL, Jensen MA, Kothiyal P, Pihl TD, Pontius J, Pot DA, Snyder EE, Srinivasan D, Mills Shaw KR, Sheth M, Davidsen T, Eley G, Ferguson ML, Yang L, Guyer MS, Ozenberger BA, Sofia HJ, Gordon Robertson A, Levine DA, Integrated genomic characterization of endometrial carcinoma, Nature 497 (2013) 67–73, 10.1038/nature12113. 23636398 PMC3704730

[73] Jiang M, Cheng Y, Wang D, Lu Y, Gu S, Wang C, Huang Y, Li Y, Transcriptional network modulated by the prognostic signature transcription factors and their long noncoding RNA partners in primary prostate cancer, EBioMedicine 63 (2021), 10.1016/j.ebiom.2020.103150. PMC7718452 33279858

[74] Montero-Ovalle W, Sanabria-Salas MC, Mesa-López de Mesa J, Varela-Ramírez R, Segura-Moreno YY, Sánchez-Villalobos SA, Nuñez-Lemus M, Serrano ML, Determination of TMPRSS2-ERG, SPOP, FOXA1, and IDH1 prostate cancer molecular subtypes in Colombian patients and their possible implications for prognosis, Cell Biol. Int. 47 (2023) 1017–1030, 10.1002/cbin.12000. 36740223

[75] Cerami E, Gao J, Dogrusoz U, Gross BE, Sumer SO, Aksoy BA, Jacobsen A, Byrne CJ, Heuer ML, Larsson E, Antipin Y, Reva B, Goldberg AP, Sander C, Schultz N, The cBio Cancer genomics portal: An open platform for exploring multidimensional Cancer genomics data, Cancer Discov. 2 (2012) 401–404, 10.1158/2159-8290.CD-12-0095. 22588877 PMC3956037

[76] Iizuka M, Susa T, Tamamori-Adachi M, Okinaga H, Okazaki T, Intrinsic ubiquitin E3 ligase activity of histone acetyltransferase Hbo1 for estrogen receptor α, Proc. Jpn. Acad. Ser. B Phys. Biol. Sci. 93 (2017) 498–510, 10.2183/pjab.93.030. PMC5713178 28769019

[77] Wang WZ, Liu HO, Wu YH, Hong Y, Yang JW, Liu YH, Bin Wu W, Zhou L, Sun LL, Xu JJ, Yun XJ, Gu JX, Estrogen receptor (ER) mediates 17-estradiol (E2)-activated expression of HBO1, J. Exp. Clin. Cancer Res. 29 (2010), 10.1186/1756-9966-29-140. PMC2989947 21040551

[78] MacPherson L, Anokye J, Yeung MM, Lam EYN, Chan YC, Weng CF, Yeh P, Knezevic K, Butler MS, Hoegl A, Chan KL, Burr ML, Gearing LJ, Willson T, Liu J, Choi J, Yang Y, Bilardi RA, Falk H, Nguyen N, Stupple PA, Peat TS, Zhang M, de Silva M, Carrasco-Pozo C, Avery VM, Khoo PS, Dolezal O, Dennis ML, Nuttall S, Surjadi R, Newman J, Ren B, Leaver DJ, Sun Y, Baell JB, Dovey O, Vassiliou GS, Grebien F, Dawson SJ, Street IP, Monahan BJ, Burns CJ, Choudhary C, Blewitt ME, Voss AK, Thomas T, Dawson MA, HBO1 is required for the maintenance of leukaemia stem cells, Nature 577 (2020) 266–270, 10.1038/s41586-019-1835-6. 31827282

[79] Yang C, Wu J, Sinha SH, Neveu JM, Zheng YG, Autoacetylation of the MYST lysine acetyltransferase MOF protein, J. Biol. Chem. 287 (2012) 34917–34926, 10.1074/jbc.M112.359356. 22918831 PMC3471714

[80] Duong MLT, Akli S, Macalou S, Biernacka A, Debeb BG, Yi M, Hunt KK, Keyomarsi K, Hbo1 is a cyclin E/CDK2 substrate that enriches breast cancer stem-like cells, Cancer Res. 73 (2013) 5556–5568, 10.1158/0008-5472.CAN-13-0013. 23955388 PMC3773499

[81] Chen TF, Hao HF, Zhang Y, Chen XY, Zhao HS, Yang R, Li P, Qiu LX, Sang YH, Xu C, Liu SX, HBO1 induces histone acetylation and is important for non-small cell lung cancer cell growth, Int. J. Biol. Sci. 18 (2022) 3313–3323, 10.7150/ijbs.72526. PMC9134900 35637972

[82] Yuan Mi Y, Ji Y, Zhang L, Yu Sun C, Bing Wei B, Jie Yang D, Yuan Wan H, Wei Qi X, Wu S, Jie Zhu L, A first-in-class HBO1 inhibitor WM-3835 inhibits castration-resistant prostate cancer cell growth in vitro and in vivo, Cell Death Dis. 14 (2023), 10.1038/s41419-023-05606-5. PMC9884225 36709328

[83] Zhang C, Zhu J, Lin H, Zhang Z, Kang B, Li F, Shan Y, Zhang Y, Xing Q, Gu J, Hu X, Cui Y, Huang J, Zhou T, Mai Y, Chen Q, Mao R, Li P, Pan G, HBO1 determines epithelial-mesenchymal transition and promotes immunotherapy resistance in ovarian cancer cells, Cell. Oncol. (2025), 10.1007/s13402-025-01055-8. PMC12238117 40227530

[84] Chen Z, Zhou L, Wang L, Kazobinka G, Zhang X, Han X, Li B, Hou T, HBO1 promotes cell proliferation in bladder cancer via activation of Wnt/β-catenin signaling, Mol. Carcinog. 57 (2018) 12–21, 10.1002/mc.22715. 28796367

[85] Di Micco P, Antolin AA, Mitsopoulos C, Villasclaras-Fernandez E, Sanfelice D, Dolciami D, Ramagiri P, Mica IL, Tym JE, Gingrich PW, Hu H, Workman P, Al-Lazikani B, canSAR: update to the cancer translational research and drug discovery knowledgebase, Nucleic Acids Res. 51 (2023) D1212–D1219, 10.1093/nar/gkac1004. 36624665 PMC9825411

[86] Avvakumov N, Côté J, The MYST family of histone acetyltransferases and their intimate links to cancer, Oncogene 26 (2007) 5395–5407, 10.1038/sj.onc.1210608. 17694081

[87] Quintela M, Sieglaff DH, Gazze AS, Zhang A, Gonzalez D, Francis L, Webb P, Conlan RS, HBO1 directs histone H4 specific acetylation, potentiating mechanotransduction pathways and membrane elasticity in ovarian cancer cells, Nanomedicine 17 (2019) 254–265, 10.1016/j.nano.2019.01.017. 30759370

[88] Trisciuoglio D, Di Martile M, Del Bufalo D, Emerging role of histone acetyltransferase in stem cells and cancer, Stem Cells Int. 2018 (2018), 10.1155/2018/8908751. PMC6311713 30651738

[89] Spasova V, Mladenov B, Rangelov S, Hammoudeh Z, Nesheva D, Serbezov D, Staneva R, Hadjidekova S, Ganev M, Balabanski L, Vazharova R, Slavov C, Toncheva D, Antonova O, Clinical impact of copy number variation changes in bladder cancer samples, Exp. Ther. Med. 22 (2021), 10.3892/etm.2021.10333. PMC8243332 34257714

[90] Cantilena S, Gasparoli L, Pal D, Heidenreich O, Klusmann J, Martens JHA, Faille A, Warren AJ, Karsa M, Pandher R, Somers K, Williams O, de Boer J, Direct targeted therapy for MLL-fusion-driven high-risk acute leukaemias, Clin. Transl. Med. 12 (2022), 10.1002/ctm2.933. PMC9214753 35730653

[91] Au YZ, Gu M, De Braekeleer E, Yu J, Ong SH, Gozdecka M, Chen X, Tzelepis K, Huntly BJP, Vassiliou G, Yusa K, KAT7 Is a Therapeutic Vulnerability of *MLL* -Rearranged Acute Myeloid Leukemia, 2020, 10.1101/2020.04.25.054049.

[92] Aryal S, Zhang Y, Wren S, Li C, Lu R, Molecular regulators of HOXA9 in acute myeloid leukemia, FEBS J. 290 (2023) 321–339, 10.1111/febs.16268. 34743404

[93] Shen K, Zhang M, Wang J, Mu W, Wang J, Wang C, Xing S, Hong Z, Xiao M, Inherited heterozygous Fanconi anemia gene mutations in a therapy-related CMML patient with a rare NUP98-HOXC11 fusion: a case report, Front. Oncol. 12 (2022), 10.3389/fonc.2022.1036511. PMC9626966 36338706

[94] Takahashi S, Kanai A, Okuda H, Miyamoto R, Komata Y, Kawamura T, Matsui H, Inaba T, Takaori-Kondo A, Yokoyama A, Hbo1-mll interaction promotes af4/enl/p-tefb-mediated leukemogenesis, Elife 10 (2021), 10.7554/eLife.65872. PMC8387021 34431785

[95] Klonou A, Chlamydas S, Piperi C, Structure, activity and function of the mll2 (Kmt2b) protein lysine methyltransferase, Life 11 (2021), 10.3390/life11080823. PMC8401916 34440566

[96] de Bruijn I, Kundra R, Mastrogiacomo B, Tran TN, Sikina L, Mazor T, Li X, Ochoa A, Zhao G, Lai B, Abeshouse A, Baiceanu D, Ciftci E, Dogrusoz U, Dufilie A, Erkoc Z, Garcia Lara E, Fu Z, Gross B, Haynes C, Heath A, Higgins D, Jagannathan P, Kalletla K, Kumari P, Lindsay J, Lisman A, Leenknegt B, Lukasse P, Madela D, Madupuri R, van Nierop P, Plantalech O, Quach J, Resnick AC, Rodenburg SYA, Satravada BA, Schaeffer F, Sheridan R, Singh J, Sirohi R, Sumer SO, van Hagen S, Wang A, Wilson M, Zhang H, Zhu K, Rusk N, Brown S, Lavery JA, Panageas KS, Rudolph JE, LeNoue-Newton ML, Warner JL, Guo X, Hunter-Zinck H, Yu TV, Pilai S, Nichols C, Gardos SM, Philip J, Kehl KL, Riely GJ, Schrag D, Lee J, Fiandalo MV, Sweeney SM, Pugh TJ, Sander C, Cerami E, Gao J, Schultz N, Analysis and visualization of longitudinal genomic and clinical data from the AACR Project GENIE Biopharma Collaborative in cBioPortal, Cancer Res. 83 (2023) 3861–3867, 10.1158/0008-5472.CAN-23-0816. 37668528 PMC10690089

[97] Wu J, Li L, Jiang G, Zhan H, Zhu X, Yang W, NCAPG2 facilitates glioblastoma cells’ malignancy and xenograft tumor growth via HBO1 activation by phosphorylation, Cell Tissue Res. 383 (2021) 693–706, 10.1007/s00441-020-03281-y. 32897418

[98] Ren W, Yang S, Chen X, Guo J, Zhao H, Yang R, Nie Z, Ding L, Zhang L, NCAPG2 is a novel prognostic biomarker and promotes Cancer stem cell maintenance in low-grade glioma, Front. Oncol. 12 (2022), 10.3389/fonc.2022.918606. PMC9309203 35898895

[99] Wu J, Li L, Jiang G, Zhan H, Zhu X, Yang W, NCAPG2 Facilitates Glioblastoma Cells’ Malignancy and Xenograft Tumor Growth Via HBO1 Activation by Phosphorylation, (n.d.). doi:10.1007/s00441-020-03281-y/Published. 32897418

[100] Latour M, Her NG, Kesari S, Nurmemmedov E, WNT Signaling as a therapeutic target for glioblastoma, Int. J. Mol. Sci. 22 (2021), 10.3390/ijms22168428. PMC8395085 34445128

[101] Wu J, Li L, Jiang G, Zhan H, Zhu X, Yang W, NCAPG2 Facilitates Glioblastoma Cells’ Malignancy and Xenograft Tumor Growth Via HBO1 Activation by Phosphorylation, (n.d.). doi:10.1007/s00441-020-03281-y/Published. 32897418

[102] Jiang S, Huang J, He H, Liu Y, Liang L, Sun X, Li Y, Cong L, Qing B, Jiang Y, NCAPG2 maintains Cancer stemness and promotes erlotinib resistance in lung adenocarcinoma, Cancers (Basel) 14 (2022), 10.3390/cancers14184395. PMC9497119 36139554

[103] Wang Q, Li Z, Zhou S, Li Z, Huang X, He Y, Zhang Y, Zhao X, Tang Y, Xu M, NCAPG2 could be an immunological and prognostic biomarker: from pan-cancer analysis to pancreatic cancer validation, Front. Immunol. 14 (2023), 10.3389/fimmu.2023.1097403. PMC9911455 36776838

[104] Bao P, Li P, Zhou X, Zhang H, You S, Xu Z, Wu Q, SMAR1 inhibits proliferation, EMT and Warburg effect of bladder cancer cells by suppressing the activity of the Wnt/β-catenin signaling pathway, Cell Cycle 22 (2023) 229–241, 10.1080/15384101.2022.2112006. 35980125 PMC9817122

[105] Wang H, Qiu Y, Zhang H, Chang N, Hu Y, Chen J, Hu R, Liao P, Li Z, Yang Y, Cen Q, Ding X, Li M, Xie X, Li Y, Histone acetylation by HBO1 (KAT7) activates Wnt/β-catenin signaling to promote leukemogenesis in B-cell acute lymphoblastic leukemia, Cell Death Dis. 14 (2023), 10.1038/s41419-023-06019-0. PMC10403501 37542030

[106] Tan T, Shi P, Abbas MN, Wang Y, Xu J, Chen Y, Cui H, Epigenetic modification regulates tumor progression and metastasis through EMT (review), Int. J. Oncol. 60 (2022), 10.3892/ijo.2022.5360. PMC9084613 35445731

[107] Zhu Q, Zhou H, Xie F, Regulation of ovarian cancer by protein post-translational modifications, Front. Oncol. 14 (2024), 10.3389/fonc.2024.1437953. PMC11638062 39678497

[108] Lili LN, Matyunina LV, Walker LD, Wells SL, Benigno BB, McDonald JF, Molecular profiling supports the role of epithelial-to-mesenchymal transition (EMT) in ovarian cancer metastasis, J. Ovarian Res. 6 (2013), 10.1186/1757-2215-6-49. PMC3726281 23837907

[109] Xu J, Lamouille S, Derynck R, TGF-В-induced epithelial to mesenchymal transition, Cell Res. 19 (2009) 156–172, 10.1038/cr.2009.5. PMC4720263 19153598

[110] Chen Y, Wang DD, Wu YP, Su D, Zhou TY, Gai RH, Fu YY, Zheng L, He QJ, Zhu H, Yang B, MDM2 promotes epithelial-mesenchymal transition and metastasis of ovarian cancer SKOV3 cells, Br. J. Cancer 117 (2017) 1192–1201, 10.1038/bjc.2017.265. 28817834 PMC5674096

[111] Zhang C, Shan Y, Lin H, Zhang Y, Xing Q, Zhu J, Zhou T, Lin A, Chen Q, Wang J, Pan G, HBO1 determines SMAD action in pluripotency and mesendoderm specification, Nucleic Acids Res. 52 (2024) 4935–4949, 10.1093/nar/gkae158. 38421638 PMC11109972

[112] Li L, Liang Y, Kang L, Liu Y, Gao S, Chen S, Li Y, You W, Dong Q, Hong T, Yan Z, Jin S, Wang T, Zhao W, Mai H, Huang J, Han X, Ji Q, Song Q, Yang C, Zhao S, Xu X, Ye Q, Transcriptional regulation of the Warburg effect in Cancer by SIX1, Cancer Cell 33 (2018) 368–385.e7, 10.1016/j.ccell.2018.01.010. 29455928

[113] Zhang X, Zhang X, Chen L, Zhao J, Raj A, Wang Y, Li S, Zhang C, Yang J, Sun D, Adipose mesenchymal stem cell-derived exosomes enhanced glycolysis through the SIX1/HBO1 pathway against oxygen and glucose deprivation injury in human umbilical vein endothelial cells, Curr. Stem Cell Res. Ther. 19 (2024) 1153–1163, 10.2174/011574888X265623230921045240. 37779410

[114] Chu Y, Jiang M, Wu N, Xu B, Li W, Liu H, Su S, Shi Y, Liu H, Gao X, Fu X, Chen D, Li X, Wang W, Liang J, Nie Y, Fan D, O-GlcNAcylation of SIX1 enhances its stability and promotes hepatocellular carcinoma proliferation, Theranostics 10 (2020) 9830–9842, 10.7150/thno.45161. 32863962 PMC7449927

[115] Kahlert C, Lerbs T, Pecqueux M, Herpel E, Hoffmeister M, Jansen L, Brenner H, Chang-Claude J, Bläker H, Kloor M, Roth W, Pilarsky C, Rahbari NN, Schölch S, Bork U, Reissfelder C, Weitz J, Aust D, Koch M, Overexpression of SIX1 is an independent prognostic marker in stage I-III colorectal cancer, Int. J. Cancer 137 (2015) 2104–2113, 10.1002/ijc.29596. 25951369

[116] Hua L, Fan L, Aichun W, Yongjin Z, Qingqing C, Xiaojian W, Inhibition of Six1 promotes apoptosis, suppresses proliferation, and migration of osteosarcoma cells, Tumour Biol. 35 (2014) 1925–1931, 10.1007/s13277-013-1258-1. 24114014

[117] Li L, Zhang X, Xu G, Xue R, Li S, Wu S, Yang Y, Lin Y, Lin J, Liu G, Gao S, Zhang Y, Ye Q, Transcriptional regulation of De novo lipogenesis by SIX1 in liver Cancer cells, Adv. Sci. (2024), 10.1002/advs.202404229. PMC11538671 39258807

[118] Wang Q, He R, Tan T, Li J, Hu Z, Luo W, Duan L, Luo W, Luo D, A novel long non-coding RNA-KAT7 is low expressed in colorectal cancer and acts as a tumor suppressor, Cancer Cell Int. 19 (2019), 10.1186/s12935-019-0760-y. PMC6390533 30858757

[119] Sun X, Hu F, Hou Z, Chen Q, Lan J, Luo X, Wang G, Hu J, Cao Z, SIX4 activates Akt and promotes tumor angiogenesis, Exp. Cell Res. 383 (2019), 10.1016/j.yexcr.2019.111495. 31301290

[120] Guo X, Li Y, Wan B, Lv Y, Wang X, Liu G, Wang P, KAT7 promoted gastric cancer progression through promoting YAP1 activation, Pathol. Res. Pract. 237 (2022), 10.1016/j.prp.2022.154020. 35868058

[121] Ritsvall O, Albinsson S, Emerging role of YAP/TAZ in vascular mechanotransduction and disease, Microcirculation 31 (2024), 10.1111/micc.12838. 38011540

[122] Cai X, Wang KC, Meng Z, Mechanoregulation of YAP and TAZ in cellular homeostasis and disease progression, Front. Cell Dev. Biol. 9 (2021), 10.3389/fcell.2021.673599. PMC8182050 34109179

[123] Hall CA, Wang R, Miao J, Oliva E, Shen X, Wheeler T, Hilsenbeck SG, Orsulic S, Goode S, Hippo pathway effector yap is an ovarian Cancer oncogene, Cancer Res. 70 (2010) 8517–8525, 10.1158/0008-5472.CAN-10-1242. 20947521 PMC2970655

[124] Jeong W, Kim S-B, Sohn BH, Park Y-Y, Park ES, Kim SC, Kim SS, Johnson RL, Birrer M, Bowtell DSL, Mills GB, Sood A, Lee J-S, Activation of YAP1 Is Associated with Poor Prognosis and Response to Taxanes in Ovarian Cancer. http://www.r, 2014. PMC4082822 24511017

[125] Zeng Q, Wang K, Zhao Y, Ma Q, Chen Z, Huang W, Effects of the acetyltransferase p300 on tumour regulation from the novel perspective of posttranslational protein modification, Biomolecules 13 (2023), 10.3390/biom13030417. PMC10046601 36979352

[126] Iizuka M, Sarmento OF, Sekiya T, Scrable H, Allis CD, Smith MM, Hbo1 links p53-dependent stress Signaling to DNA replication licensing, Mol. Cell. Biol. 28 (2008) 140–153, 10.1128/mcb.00662-07. 17954561 PMC2223294

[127] Lamb R, Lehn S, Rogerson L, Clarke RB, Landberg G, Cell cycle regulators cyclin D1 and CDK4/6 have estrogen receptor-dependent divergent functions in breast cancer migration and stem cell-like activity, Cell Cycle 12 (2013) 2384–2394, 10.4161/cc.25403. 23839043 PMC3841318

[128] Zhang C, Zhu J, Lin H, Zhang Z, Kang B, Li F, Shan Y, Zhang Y, Xing Q, Gu J, Hu X, Cui Y, Huang J, Zhou T, Mai Y, Chen Q, Mao R, Li P, Pan G, HBO1 determines epithelial-mesenchymal transition and promotes immunotherapy resistance in ovarian cancer cells, Cell. Oncol. (2025), 10.1007/s13402-025-01055-8. PMC12238117 40227530

[129] Löhr K, Möritz C, Contente A, Dobbelstein M, p21/CDKN1A mediates negative regulation of transcription by p53, J. Biol. Chem. 278 (2003) 32507–32516, 10.1074/jbc.M212517200. 12748190

[130] Wright DG, Marchal C, Hoang K, Ankney JA, Nguyen ST, Rushing AW, Polakowski N, Miotto B, Lemasson I, Human T-Cell Leukemia Virus Type-1-Encoded Protein HBZ Represses p53 Function by Inhibiting the Acetyltransferase Activity of p300/CBP and HBO1, n.d. www.impactjournals.com/oncotarget/. 10.18632/oncotarget.6424 PMC4811490 26625199

[131] Jackson JG, Pereira-Smith OM, p53 is preferentially recruited to the promoters of growth arrest genes *p21* and *GADD45* during replicative senescence of Normal human fibroblasts, Cancer Res. 66 (2006) 8356–8360, 10.1158/0008-5472.CAN-06-1752. 16951143

[132] Tang Y, Zhang H, Liu J, Zhao J, He Q, Pan C, Zheng K, Wan L, Lactylation and human disease, Expert Rev. Mol. Med. (2025), 10.1017/erm.2025.3. PMC11879378 39895568

[133] Yu X, Yang J, Xu J, Pan H, Wang W, Yu X, Shi S, Histone lactylation: from tumor lactate metabolism to epigenetic regulation, Int. J. Biol. Sci. 20 (2024) 1833–1854, 10.7150/ijbs.91492. PMC10929197 38481814

[134] Lv M, Huang Y, Chen Y, Ding K, Lactylation modification in cancer: mechanisms, functions, and therapeutic strategies, Exp. Hematol. Oncol. 14 (2025), 10.1186/s40164-025-00622-x. PMC11889934 40057816

[135] Niu Z, Chen C, Wang S, Lu C, Wu Z, Wang A, Mo J, Zhang J, Han Y, Yuan Y, Zhang Y, Zang Y, He C, Bai X, Tian S, Zhai G, Wu X, Zhang K, HBO1 catalyzes lysine lactylation and mediates histone H3K9la to regulate gene transcription, Nat. Commun. 15 (2024), 10.1038/s41467-024-47900-6. PMC11053077 38670996

[136] Wang X, Liu X, Xiao R, Fang Y, Zhou F, Gu M, Luo X, Jiang D, Tang Y, You L, Zhao Y, Histone lactylation dynamics: unlocking the triad of metabolism, epigenetics, and immune regulation in metastatic cascade of pancreatic cancer, Cancer Lett. 598 (2024), 10.1016/j.canlet.2024.217117. 39019144

[137] Niu Z, Chen C, Wang S, Lu C, Wu Z, Wang A, Mo J, Zhang J, Han Y, Yuan Y, Zhang Y, Zang Y, He C, Bai X, Tian S, Zhai G, Wu X, Zhang K, HBO1 catalyzes lysine lactylation and mediates histone H3K9la to regulate gene transcription, Nat. Commun. 15 (2024), 10.1038/s41467-024-47900-6. PMC11053077 38670996

[138] Song B, Liu XS, Rice SJ, Kuang S, Elzey BD, Konieczny SF, Ratliff TL, Hazbun T, Chiorean EG, Liu X, Plk1 phosphorylation of Orc2 and Hbo1 contributes to gemcitabine resistance in pancreatic cancer, Mol. Cancer Ther. 12 (2013) 58–68, 10.1158/1535-7163.MCT-12-0632. 23188630 PMC3732037

[139] Jiang Q, Zhang N, Li X, Hou W, Zhao X-Q, Liu L, Dl-3-N-butylphthalide presents anti-Cancer activity in lung Cancer by targeting PD-1/PD-L1 Signaling, Cancer Manag. Res. 13 (2021) 8513–8524, 10.2147/CMAR.S333416. 34795530 PMC8594621

[140] Bai Z, Xia X, Lu J, MicroRNA-639 is down-regulated in hepatocellular carcinoma tumor tissue and inhibits proliferation and migration of human hepatocellular carcinoma cells through the KAT7/Wnt/b-catenin pathway, Med. Sci. Monit. 26 (2020), 10.12659/MSM.919241. PMC6988476 31955177

[141] Sakamoto KM, Kim KB, Kumagai A, Mercurio F, Crews CM, Deshaies RJ, Protacs: chimeric molecules that target proteins to the Skp1–Cullin–F box complex for ubiquitination and degradation, Proc. Natl. Acad. Sci. 98 (2001) 8554–8559, 10.1073/pnas.141230798. 11438690 PMC37474

[142] Lu J, Qian Y, Altieri M, Dong H, Wang J, Raina K, Hines J, Winkler JD, Crew AP, Coleman K, Crews CM, Hijacking the E3 ubiquitin ligase Cereblon to efficiently target BRD4, Chem. Biol. 22 (2015) 755–763, 10.1016/j.chembiol.2015.05.009. 26051217 PMC4475452

[143] Mancarella C, Morrione A, Scotlandi K, PROTAC-based protein degradation as a promising strategy for targeted therapy in sarcomas, Int. J. Mol. Sci. 24 (2023) 16346, 10.3390/ijms242216346. 38003535 PMC10671294

